# A Para-Substituted 2-Phenoxy-1,10-Phenanthroline Ligand for Lanthanide Sensitization: Asymmetric Coordination and Enhanced Emission from Eu^3+^, Tb^3+^, Sm^3+^ and Dy^3+^ Complexes

**DOI:** 10.3390/molecules30173548

**Published:** 2025-08-29

**Authors:** Joana Zaharieva, Vladimira Videva, Mihail Kolarski, Rumen Lyapchev, Bernd Morgenstern, Martin Tsvetkov

**Affiliations:** 1 Laboratory of Chemistry of Rare-Earth Elements, Faculty of Chemistry and Pharmacy, Sofia University “St. Kliment Ohridski”, 1164 Sofia, Bulgaria; 2 Faculty of Chemistry and Pharmacy, Sofia University “St. Kliment Ohridski”, 1164 Sofia, Bulgaria; ohtvv@chem.uni-sofia.bg (V.V.); mkolarski@uni-sofia.bg (M.K.); ohrl@chem.uni-sofia.bg (R.L.); 3 Institute of Optical Materials and Technologies, Bulgarian Academy of Sciences, 1113 Sofia, Bulgaria; 4 Inorganic Solid State Chemistry, Saarland University, Campus Geb. C4 1, 66123 Saarbrücken, Germany; bernd.morgenstern@uni-saarland.de

**Keywords:** lanthanide complex, luminescence properties, crystal structure, 1,10-phenanthroline derivate

## Abstract

A para-substituted 1,10-phenanthroline ligand, 2-(4-methylphenoxy)-1,10-phenanthroline (L24), was synthesized and structurally characterized. Complexes with Eu^3+^, Tb^3+^, Sm^3+^, and Dy^3+^ were obtained in a 2:1 ligand-to-metal ratio and analyzed using single-crystal x-ray diffraction, photoluminescence spectroscopy, and TD-DFT calculations. Coordination via the phenanthroline nitrogen atoms, combined with steric asymmetry from the para-methylphenoxy group, induces low-symmetry environments favorable for electric-dipole transitions. Excited-state lifetimes reached 2.12 ms (Eu^3+^) and 1.12 ms (Tb^3+^), with quantum yields of 42% and 68%, respectively. The triplet-state energy of L24 (22,741 cm^−1^) aligns well with emissive levels of Eu^3+^ and Tb^3+^, consistent with Latva’s criterion. Fluorescence titrations indicated positively cooperative complexation, with association constants ranging from 0.60 to 1.67. Stark splitting and high ^5^D_0_ → ^7^F_2_/^7^F_1_ intensity ratios (R_2_ = 6.25) confirm the asymmetric coordination field. The para-methylphenoxy substituent appears sufficient to lower coordination symmetry and strengthen electric-dipole transitions, offering a controlled route to enhance photoluminescence in Eu^3+^ and Tb^3+^ complexes.

## 1. Introduction

Lanthanide coordination compounds remain central to the development of advanced photonic materials due to their sharp emission lines, long excited-state lifetimes, and high photostability. These properties support applications in time-resolved bioimaging, optical sensing, and solid-state lighting [1]. The luminescence arises from 4f–4f transitions, which are parity-forbidden and shielded from environmental perturbations. While this shielding confers stability, it also results in intrinsically weak absorption.

To overcome the low molar absorptivity of lanthanide ions, sensitization via organic ligands—the so-called antenna effect—is widely employed. Upon ligand excitation, intersystem crossing populates the triplet state, which can transfer energy to the lanthanide’s emissive level, provided the energetic alignment is suitable. In rigid coordination environments, Dexter-type mechanisms involving short-range exchange interactions often dominate the energy transfer process [2,3,4,5].

For efficient sensitization, the energy gap between the ligand triplet state and the lanthanide emissive level should typically fall within 2500–4000 cm^−1^—a range defined by Latva’s criterion [3,5]. Ligands based on 1,10-phenanthroline (phen) are particularly suited to this task due to their high triplet-state energies, rigidity, and predictable chelating geometry [2]. A wide range of 2-substituted or 2,9-disubstituted phenanthrolines have been reported as antenna ligands, typically introducing hydrophobicity, steric bulk, or additional binding sites [6].

However, these substituted phenanthroline systems are often symmetric and designed to stabilize predictable coordination environments. As a result, many phen-based complexes exhibit centrosymmetric geometries [7], which are not optimal for electric-dipole transitions. Recent reports have emphasized the importance of asymmetric ligand fields in promoting hypersensitive transitions such as Eu^3+ 5^D_0_ → ^7^F_2_ and Sm^3+ 4^G_5⁄2_ → ^6^H_7⁄2_, leading to stronger emission in low-symmetry environments [8].

Despite this, para-substitution at the aryloxy group in position 2 of the phen core remains underexplored as a strategy for introducing unsymmetry. In particular, small, non-coordinating electron-donating groups—such as methyl—could serve as structural modulators that subtly lower symmetry without disturbing the chelating behavior of the ligand. The absence of sterically or electronically disruptive effects makes such substituents ideal for isolating the impact of unsymmetry alone.

Here we introduce 2-(4-methylphenoxy)-1,10-phenanthroline, a 2-phenoxy-1,10-phenanthroline ligand designed to explore the effect of mild peripheral asymmetry on coordination geometry and luminescence. Unlike bulkier substituents or extended π-conjugation systems, the para-methylphenoxy group is expected to preserve ligand rigidity and solubility while modulating the local ligand field around the metal center. This makes the ligand a minimal yet targeted modification of the classic phen scaffold.

We selected Eu^3+^ and Tb^3+^ as prototypical emissive ions for evaluating ligand-to-metal energy transfer, given their high sensitivity to ligand symmetry (Eu^3+^) and high quantum efficiency (Tb^3+^). Sm^3+^ and Dy^3+^ were included as lower-intensity, but spectrally informative emitters relevant for sensing, bioimaging, and magneto-optical applications [8]. Complexes were synthesized in 2:1 stoichiometry and characterized by single-crystal X-ray diffraction, steady-state and time-resolved photoluminescence spectroscopy, and TD-DFT calculations. The results show that even modest peripheral substitution is sufficient to disrupt centrosymmetry, influence sensitization efficiency, and tune emission profiles across the lanthanide series.

## 2. Results and Discussion

### 2.1. Structural Characterization by FTIR

**Figure 1:**
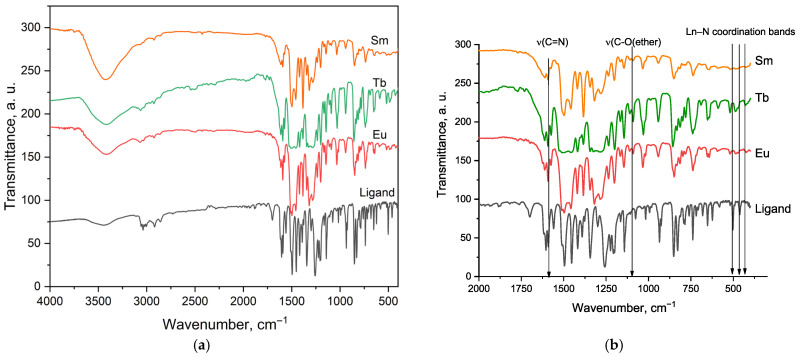
FTIR spectra of the free ligand L24 and its Sm^3+^, Tb^3+^, and Eu^3+^ complexes: (**a**) full 4000–400 cm^−1^ range and (**b**) expanded 2000–400 cm^−1^ region with highlighted ν(C=N) and low-frequency Ln–N coordination bands.

The FTIR spectrum of the free ligand L24 (Figure 1) shows clear vibrational features consistent with its structure. The strong band at about 1585 cm^−1^ is assigned to the ν(C=N) stretching of the phenanthroline ring, in line with similar systems [9]. Multiple absorptions between 1300 and 1000 cm^−1^ correspond to ν(C–N), ν(C–C), and ν(C–O) vibrations, the latter arising from the ether group at the 1′-position [10]. Signals below 800 cm^−1^ are associated with out-of-plane aromatic deformations, reflecting the rigidity of the ligand backbone [10]. Upon coordination with Eu^3+^, Tb^3+^, Sm^3+^, and Dy^3+^, several changes are observed. The ν(C=N) band shifts by 5–8 cm^−1^ to lower frequencies, indicating binding of the phenanthroline nitrogen atoms to the metal [11]. Bands in the 1200–1000 cm^−1^ region also shift and change in intensity, suggesting that the ether oxygen contributes to coordination [12]. In the 600–400 cm^−1^ range, new bands appear and some of the original ones weaken or move, reflecting metal–nitrogen and metal–oxygen stretching modes [12]. The positions and relative intensities of these low-frequency vibrations differ between the Eu, Tb, and Sm complexes.

### 2.2. Crystal Structure

Suitable for single crystal diffraction crystals from the ligand and the Dy(III) complex were obtained by slow evaporation of acetonitrile solutions. The experimental details of the single crystal diffraction measurements as well as the corresponding CCDC deposition numbers are summarized in Table S1. These data can be obtained free of charge via https://www.ccdc.cam.ac.uk/structures/, accessed on 26 August 2025 (or from the Cambridge Crystallographic Data Centre, 12, Union Road, Cambridge CB2 1EZ, UK; fax: +44 1223 336033).

**Figure 2:**
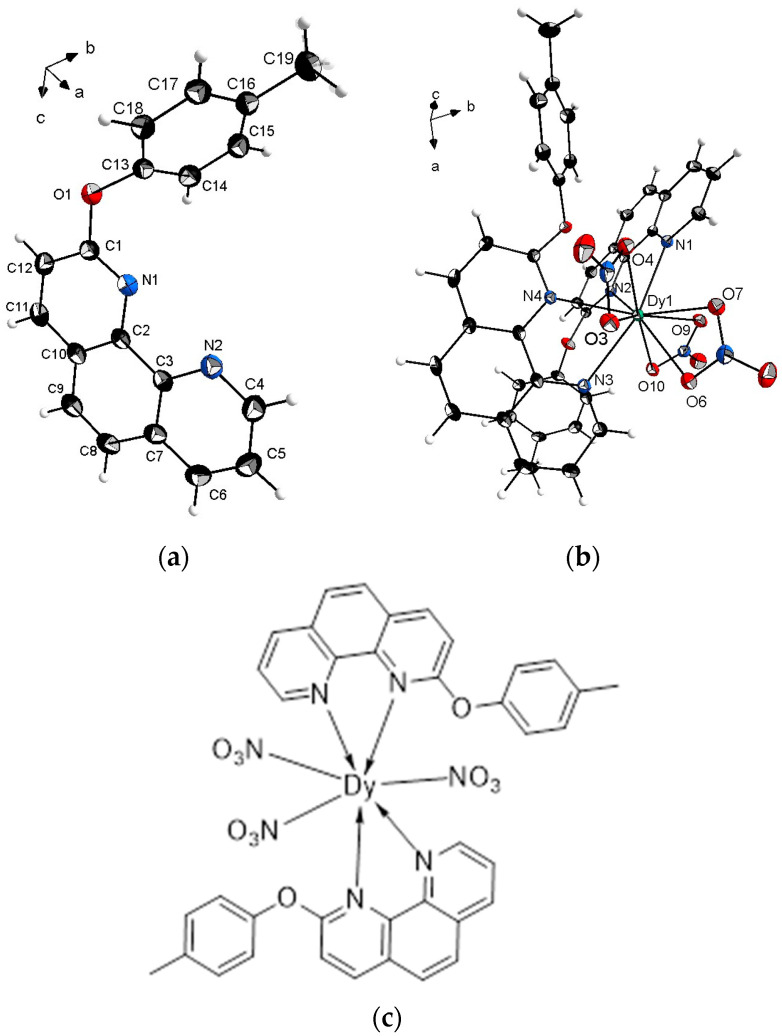
Molecular structure of (**a**) the ligand; (**b**) its Dy-complex and (**c**) a schematic representation of the [Ln(L24)_2_(NO_3_)_3_] complexes (Ln = Eu, Tb, Sm, Dy).

The 2-(4-methylphenoxy)-1,10-phenanthroline ligand crystallizes in the orthorhombic, *Pbca* space group which is relatively high symmetry for such organic molecules. The asymmetric unit contains one molecule (Figure 2a) while the unit cell contains eight ligand molecules. The crystal structure of the ligand is additionally stabilized by weak intermolecular hydrogen bonds (Figure S2a).

**Figure 3:**
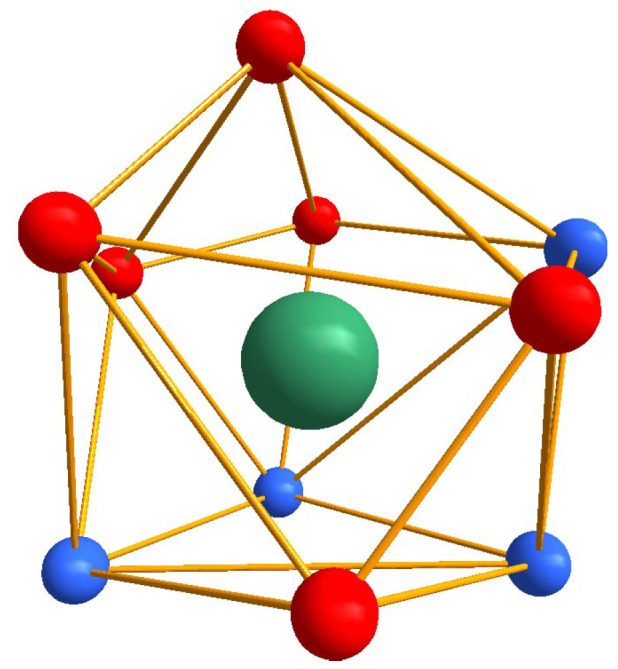
Shape of the coordination polyhedral of the Dy(III).

The Dy-complex crystallizes in the triclinic *P*$\bar{1}$ space group with one mononuclear molecule in the asymmetric unit (Figure 2b). For clarity, Figure 2c shows a schematic molecular structure of the [Ln(L24)_2_(NO_3_)_3_] complexes (Ln = Eu, Tb, Sm, Dy), illustrating the coordination of two ligands and three bidentate nitrate anions to the lanthanide center. The central metal ion is 10 coordinated (Figure 3) by four nitrogen atoms from the two 2-(4-methylphenoxy)-1,10-phenanthroline and the coordination is sphere is completed by six oxygen atoms from three nitrate ions.

Interestingly, upon complexation, the H-bond holding the adjacent organic molecules in the crystals structure is broken and a new bond is formed between the adjacent complex molecules (Figure S2b). This is related to the structural reconfiguration of the crystal structure of the ligand upon complexation related to lowering the symmetry from *Pbca* to *P*$\bar{1}$. This is most likely due to the size difference between the two ligand and complex molecules. The formation of the complex also leads to closing of the plane angle between the planes of the 1,10-phenanthroline and the 2-(4-methylphenoxy) species from 65.09(5)° to 62.47(8)° for the ligand and the complex, respectively. This is linked to slight rotation through the oxygen-bridge between the two entities; the torsion angle between the two entitles through the oxygen in the ligand is 57.55(2)°, while in the complex the angle is 45.6(2)°.

Shape of the polyhedral is important feature very often governing the luminescence properties of the lanthanide ions. Shape of the polyhedral is evaluated by continuos shape measurements using the Shape 2.1 program (Table S2) [13]. According to the results the polyhedral shape around the central metal ion can be best described as the low symmetry high disordered metabidiminished icosahedron (Johnson solid J62, symmetry *C_2v_*).

Since we were not able to obtain single crystals of the other complexes suitable for measurements, their powdered (polycrystalline) forms were characterized by powder x-ray diffraction (Figure S1). As seen on the figure, the powder diffraction of the Dy-complex in powdered form perfectly matches the simulated data from the single crystal diffraction. Furthermore, the powder diffraction patterns of the other complexes closely resemble the one of Dy, confirming that the other complexes are isostructural to it. The slight differences in the XRD patterns are due to preferred orientation of the crystals in the powdered samples and unit cell parameters, which is in good agreement with the change of the Ln(III) size from Sm to Dy.

### 2.3. Photophysical Properties

#### 2.3.1. Electronic Spectra

**Figure 4:**
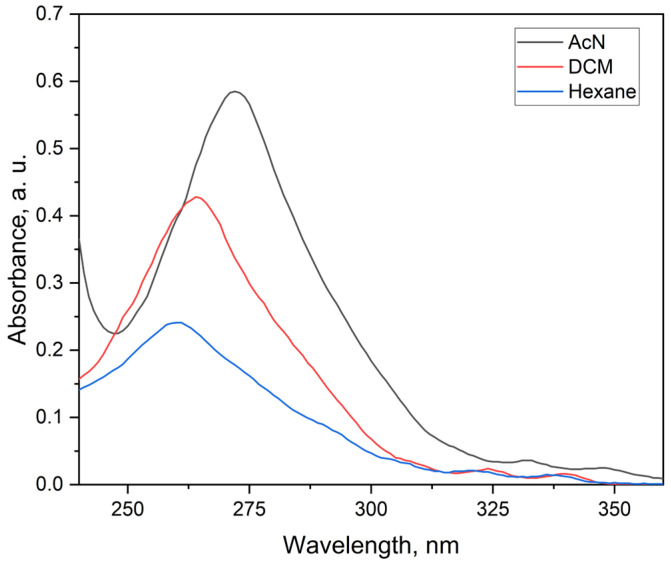
UV–Vis absorption spectra of the ligand in acetonitrile (AcN), dichloromethane (DCM), and hexane at 1.33 × 10^−7^ mol·L^−1^.

The UV–Vis absorption spectrum of the ligand (Figure 4) has a maximum of 260 nm with a molar absorptivity of up to 4.5 × 10^6^ L·mol^−1^·cm^−1^ at very low concentrations (1.33 × 10^−7^ M). At higher concentrations, this value decreases (e.g., 2.7 × 10^4^ L·mol^−1^·cm^−1^ at 1.00 × 10^−5^ M) due to inner filter effects and aggregation, which is typical for planar, conjugated ligands with strong π–π transitions [2,14].

To evaluate solvent effects, spectra were also measured in dichloromethane (DCM) and hexane at the same low concentration. The molar absorptivity followed the trend AcN > DCM > hexane, with values of 3.38 × 10^6^ and 1.88 × 10^6^ L·mol^−1^·cm^−1^, respectively. This behavior reflects moderate positive solvatochromism. The phenanthroline nitrogen atoms and ether oxygen provide sites for dipole–dipole and donor–acceptor interactions with polar solvents, which stabilize the excited state and cause a small bathochromic shift of the absorption maximum (260–275 nm). At higher concentrations in DCM and hexane, turbidity and light scattering interfered with spectral recording, suggesting reduced solubility or aggregation. These features are in line with related 2-aryl-1,10-phenanthroline ligands [15,16]. The slightly higher absorptivity of L24 may result from its increased planarity or the weak electron-donating effect of the para-methyl substituent, improving orbital overlap and transition intensity. These characteristics make L24 an efficient light-absorbing antenna in lanthanide complexes.

#### 2.3.2. Emission Spectra at Room Temperature

**Figure 5:**
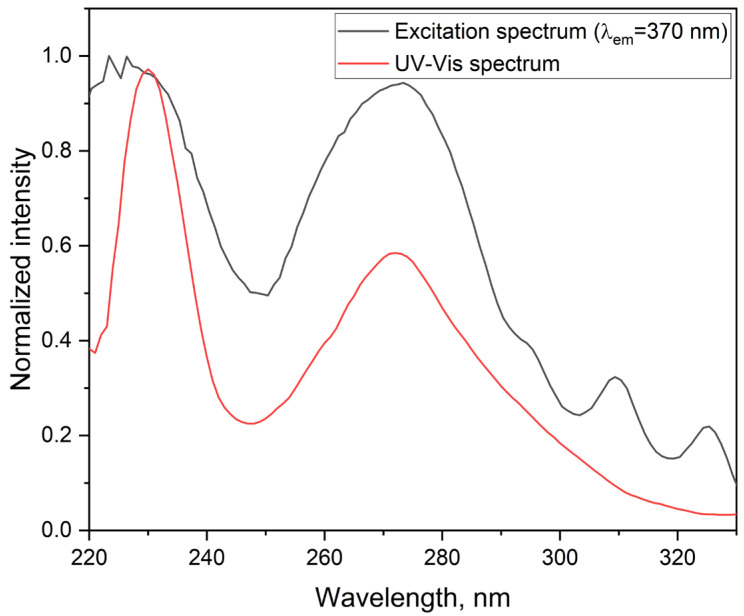
Comparison of the fluorescence excitation spectrum and UV–Vis absorption spectrum of the ligand in acetonitrile (1.0 × 10^−5^ mol·L^−1^) at room temperature.

The fluorescence excitation spectrum of the ligand was measured in acetonitrile at room temperature with the emission monitored at 370 nm (Figure 5). Two dominant excitation bands were observed near 250 nm and 295 nm, associated with π → π* transitions in the extended aromatic framework. Additional, weaker features between 310 and 345 nm likely arise from vibronic coupling or minor excited-state conformations. The close resemblance between the excitation and UV–Vis absorption spectra confirms that the fluorescence originates from the same excited-state manifold.

The notable Stokes shift, defined as the difference between the absorption maximum at 270 nm and the emission maximum at 370 nm, reflects substantial electronic reorganization and solvent stabilization—a behavior characteristic of conjugated systems in polar media.

**Figure 6:**
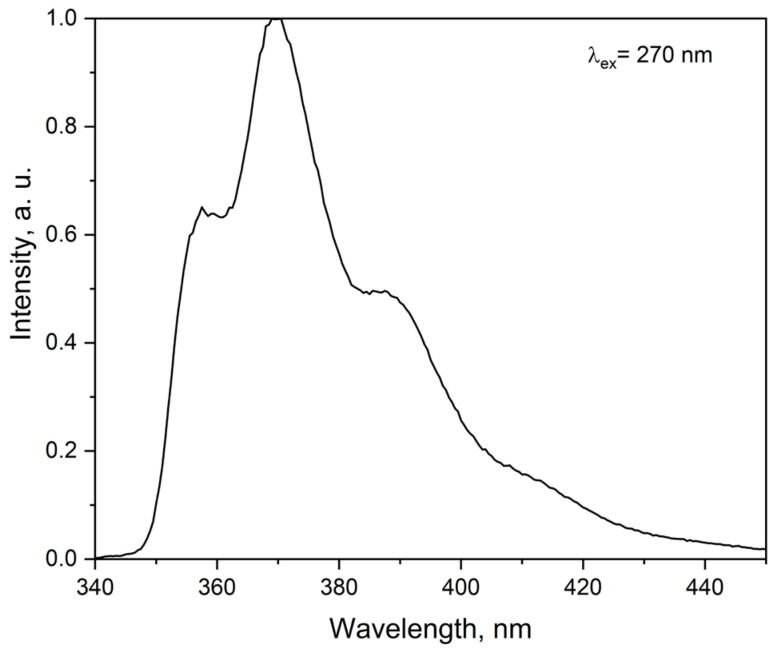
Fluorescence emission spectrum of the ligand in acetonitrile (1.0 × 10^−5^ mol·L^−1^) at room temperature.

The emission characteristics (Figure 6) of ligand in acetonitrile are consistent with fluorescence from a π–π* excited state, typical for conjugated aromatic ligands. The structured excitation and emission bands—with defined maxima and vibronic features—reflect the rigidity and planarity of the phenanthroline core extended by the para-substituted aryl group. These spectral features support the presence of a stable excited-state configuration and indicate that the ligand can act as an effective sensitizer in energy transfer to lanthanide ions.

**Figure 7:**
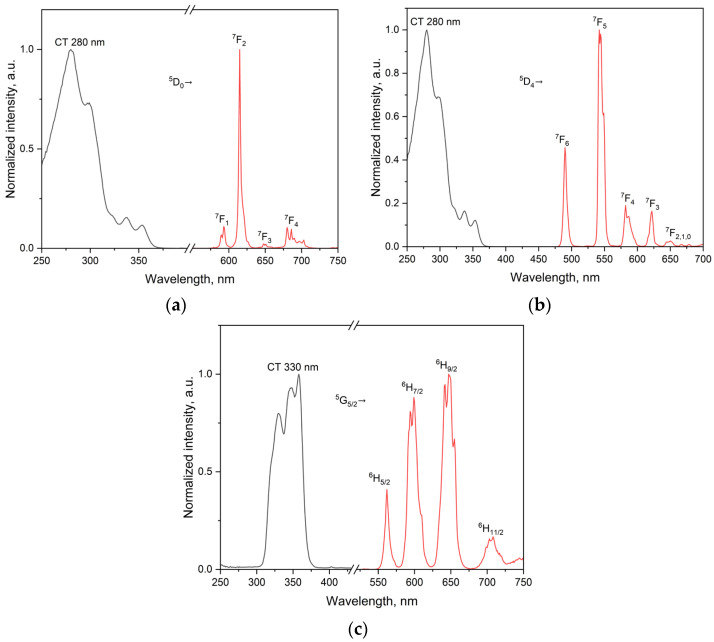
Room-temperature emission spectra (red) and excitation spectra (black) of (**a**) Eu, (**b**) Tb, and (**c**) Sm complexes in acetonitrile. Emission spectra were recorded with λ_ex_ = 295 nm for Eu^3+^ and Tb^3+^ complexes and λ_ex_ = 330 nm for the Sm^3+^ complex.

The emission spectra (red) and excitation spectra (black) of the complexes with Eu^3+^, Tb^3+^ and Sm^3+^ were recorded in acetonitrile at room temperature (Figure 7), while additional emission spectra were obtained in the solid state and in frozen solution at 77 K (Figure S3). In all cases, the characteristic f–f transitions are observed, with the degree of spectral resolution depending on both temperature and physical state.

For the Eu complex, the ^5^D_0_ → ^7^F_2_ transition dominates the spectrum in acetonitrile at 298 K, appearing as a broad, intense band with limited structure. In the solid state, resolution improves noticeably, and individual transitions become distinguishable even at room temperature. Upon cooling to 77 K (Figure S3a), full splitting is observed, including a weak but distinct ^5^D_0_ → ^7^F_0_ line, which is due to the *C_2v_* symmetry around the metal ion as proven by the continuous shape measurements [5]. The intensity distribution across the ^7^F_J_ levels is consistent with a low-symmetry coordination field that is retained across environments.

The Tb complex exhibits a similar behavior. In solution, the ^5^D_4_ → ^7^F_5_ transition is already partially structured, and further splitting appears in the solid-state spectrum. Upon cooling, the ^7^F_6_, ^7^F_5_ and ^7^F_4_ transitions are clearly resolved into multiple components, as expected for an asymmetric field without an inversion center, comparable to the europium complex.

The spectrum of the Sm complex displays the typical ^4^G_5/2_ → ^6^H_J_ transitions, which become progressively more defined from solution to the solid state and then at 77 K (Figure S3c). At room temperature, the bands are broadened but transitions to ^6^H_7⁄2_ and ^6^H_9⁄2_ remain recognizable. Upon cooling, especially in the ^6^H_11⁄2_ region, multiple components emerge clearly. Despite the lower intensity, the structure of the spectrum confirms the preservation of a low-symmetry coordination environment.

The Dy complex emits weakly, with the ^4^F_9⁄2_ → ^6^H_15⁄2_ transition around 575 nm being the most prominent. In solution, the spectrum is only partially structured and improves slightly in the solid state (Figure S3h). At 77 K, splitting becomes clear, and transitions to ^6^H_13⁄2_ and ^6^H_11⁄2_ gain definition. Although the overall intensity is low, the shape of the emission bands consists of an asymmetric ligand field, like the other complexes.

The spectral data show that the unsymmetry imposed by the ligand is preserved for all studied lanthanides, regardless of medium. In solution, it is partially masked by thermal and solvation effects but becomes clearly revealed either in the solid state or at low temperature through structured emission and resolved multiples.

In solution at room temperature, the spectral resolution is reduced due to dynamic solution and thermal broadening. The coordination environment around the metal centers remains asymmetric, but the spectral features are partially obscured by vibrational and solvent-related effects. In contrast, the solid state provides a more rigid coordination geometry, allowing better definition of individual transitions even at 298 K. The difference between solution and solid-state spectra highlights the role of solvation in modulating the observable impact of ligand field asymmetry.

**Figure 8:**
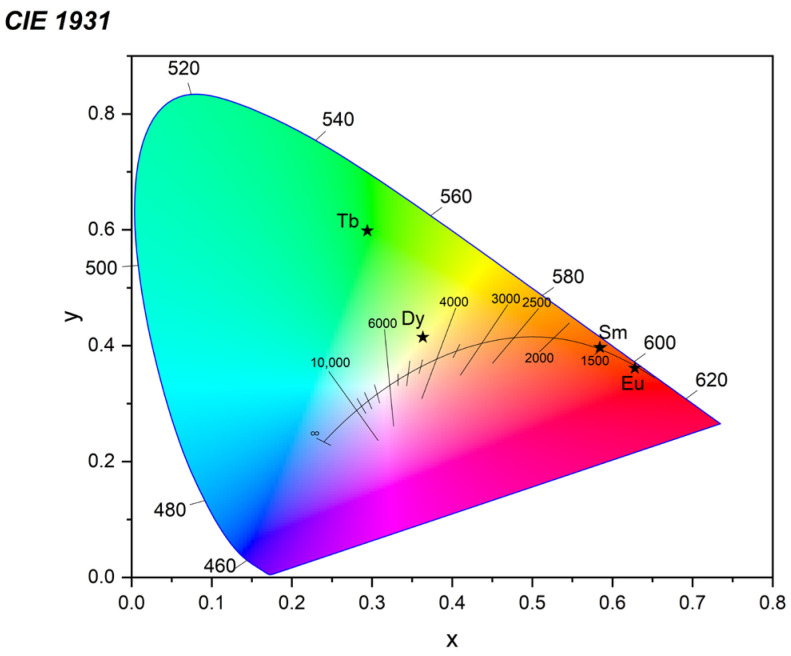
CIE 1931 chromatogram of the studied complexes where star symbols show the exact position of the related Ln-complexes.

The CIE 1931 chromatograms of all complexes in solid state are shown on Figure 8. The obtained x and y coordinates, color purity, and McCamy’s correlated color temperature (CCT) are summarized in Table S3. As seen from the data, the Eu and Sm complexes emit high purity red-orange light (color purity of Eu–96.9%, Sm–94.6%). The Tb complex expectedly emits in the yellow-green region with color purity of 69.5%. The Dy complex emits in the white region with color purity of 45.5% due to the 1:2 ratio of ^6^H_15/2_:^6^H_13/2_ emission lines.

#### 2.3.3. Spectral Deconvolution and Band Assignment

**Figure 9:**
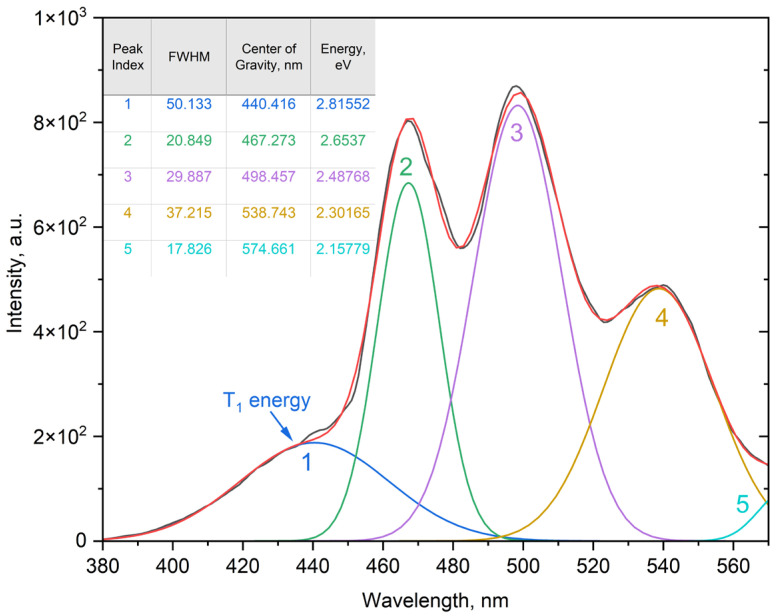
Phosphorescence spectrum of the Gd-ligand in acetonitrile at 77 K. The structured band at 467–499 nm, deconvoluted into five Gaussian components, corresponds to the T_1_ → S_0_ transition.

The phosphorescence spectrum recorded at 77 K in acetonitrile exhibits a structured emission band with an onset at ~467 nm (21,400 cm^−1^) and a maximum at 499 nm, corresponding to the T_1_ → S_0_ transition (Figure 9). Deconvolution of this band required five Gaussian components, indicative of a pronounced vibronic progression. This profile can be interpreted either as the presence of several closely spaced triplet states or as a single triplet state with strong vibronic coupling. Time-dependent DFT calculations (TDA-TDDFT at the ωB97XD/6-31G(d) level) support the latter scenario, predicting a single low-lying π–π triplet state localized on the conjugated phenanthroline core, which is typical for this class of ligands [17]. The chosen computational protocol (ωB97X-D/6-31G(d) with MWB52 ECP for Dy) has been successfully applied for lanthanide–phenanthroline systems, reproducing experimental bond lengths within 0.02–0.05 Å and coordination angles within 1–2° [18]. Geometry optimizations started from the crystallographic structure of the Dy-complex. The experimentally derived triplet-state energy of 2.82 eV (22,741 cm^−1^) is in excellent agreement with the calculated value of 2.92 eV (23,555 cm^−1^).

**Figure 10:**
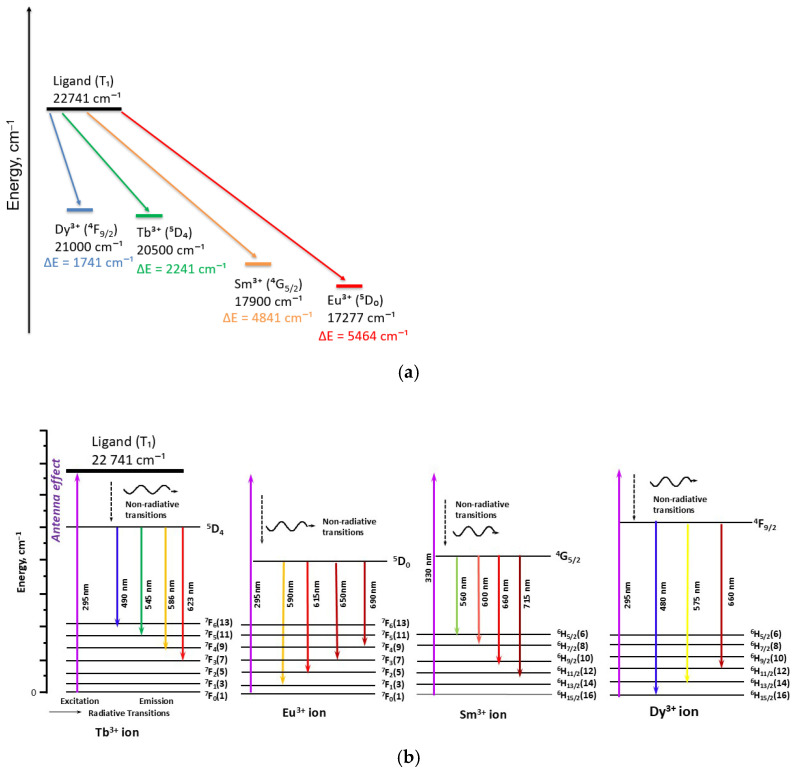
(**a**) Energy-level alignment between the triplet state of ligand and emissive levels of selected Ln^3+^ ions and (**b**) schematic energy diagrams of Eu^3+^, Tb^3+^, Sm^3+^, and Dy^3+^ complexes in a C_2_v ligand field, illustrating the antenna effect and main emission transitions (numbers of Stark sublevels indicated in parentheses).

To evaluate the influence of metal coordination on the triplet-state energy, TD-DFT calculations were extended to selected Eu^3+^, Tb^3+^, Sm^3+^, and Dy^3+^ complexes (Table 1). Coordination to Eu^3+^ and Sm^3+^ results in a moderate stabilization of the triplet state (−0.10 eV), whereas Tb^3+^ has a negligible impact. In contrast, Dy^3+^ induces a pronounced destabilization (+0.24 eV), highlighting the sensitivity of the ligand’s excited-state landscape to variations in ligand-field strength and specific metal–ligand interactions. The relative energy alignment between the ligand T_1_ level and the emissive f-levels of the lanthanide ions is depicted in Figure 10a, while Figure 10b illustrates the Stark splitting of the Eu^3+^, Tb^3+^, Sm^3+^, and Dy^3+^ emitting states within the C_2_v ligand field of the complexes.

The calculated energy gaps (ΔE) between the ligand triplet and the emissive levels of the lanthanide ions range from 1741 cm^−1^ for Dy^3+^ to 5464 cm^−1^ for Tb^3+^, with intermediate values for Eu^3+^ and Sm^3+^. According to the Latva criterion [3], which requires a minimum ΔE of ~3000 cm^−1^ to effectively suppress thermal back-transfer, these results confirm that the ligand is energetically well-suited for sensitization of Eu^3+^, Tb^3+^, and Sm^3+^. In contrast, the small ΔE for Dy^3+^ places it near the threshold where back-energy transfer and multiphonon relaxation become competitive with radiative decay, providing a rationale for its markedly lower quantum yield and shorter excited-state lifetime. This interpretation is consistent with previous reports demonstrating that energy gaps below ~2000 cm^−1^ strongly favor non-radiative deactivation over luminescence [16]. Similar correlations between the ligand triplet-state energy and the emissive levels of Eu^3+^, Tb^3+^, and Sm^3+^ have also been reported for other antenna ligands. Subsequent studies on substituted phenanthrolines and bipyridyl derivatives [17,19,20] have confirmed that too small a gap promotes non-radiative processes at the expense of luminescence. These observations put our findings for the [Ln(L24)_2_(NO_3_)_3_] complexes in a broader context and show that they are in line with established trends.

#### 2.3.4. Photophysical Parameters Derived from Emission Studies

**Figure 11:**
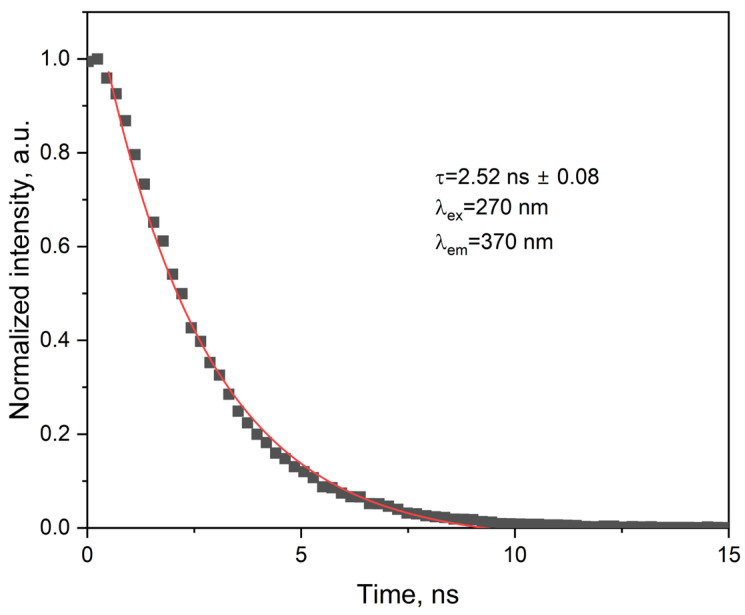
Time-resolved fluorescence decay of the ligand.

The fluorescence decay of the ligand was recorded in acetonitrile at room temperature and is presented in Figure 11. The profile follows a monoexponential function, described by the equation I(t) = A·exp(−t/τ) + y_0_, with a fitted lifetime (τ) of 2.52 ± 0.08 ns. The quality of the fit (R^2^ = 0.9905) confirms the presence of a single emissive species. This value is consistent with reported lifetimes for related 1,10-phenanthroline derivatives, which typically range between 2 and 3 ns under comparable conditions [21]. The monoexponential decay suggests that the excited-state deactivation occurs predominantly through a single emissive state. While minor effects from solvent relaxation or conformational dynamics cannot be excluded, their influence appears negligible under the conditions studied. The substitution at the 2-position with a 4-methylphenoxy group does not significantly alter the decay kinetics.

The photophysical properties of Eu, Tb, and Sm complexes were investigated by time-resolved luminescence measurements in acetonitrile (AcN), dichloromethane (DCM), and in the solid state at room temperature (Table 2). The data reveal distinct solvent- and ion-dependent variations in lifetime behavior, reflecting both the sensitization efficiency of chosen ligand towards the different lanthanide ions and the coordination environment of the complexes. The latter may vary between solution and solid state due to possible coordination of solvent molecules, such as acetonitrile, in solution. This aspect is discussed further in the context of solvent-dependent emission behavior. For Eu, the luminescence lifetime ranges from 1.31 ms in the solid state to 2.12 ms in acetonitrile, indicating efficient ligand-to-metal energy transfer. The longer lifetime in AcN compared to DCM (2.12 vs. 1.80 ms) suggests enhanced stabilization of the excited state in the more polar solvent. A moderate reduction in the solid-state value may be attributed to vibrational quenching or non-radiative pathways associated with lattice dynamics.

Compared to structurally related europium complexes, such as [Eu(PEP)_2_(NO_3_)_3_] (1.39 ms in AcN, 1.67 ms in DCM) [7], Eu-complex shows comparable or slightly improved performance in solution. This may be attributed to increased coordination asymmetry and favorable ligand field effects introduced by the para-methylphenoxy substituent at the 2-position, which likely disrupts inversion symmetry and enhances the radiative ^5^D_0_ → ^7^F_2_ transition.

In contrast to Eu, the Tb-complex displays lifetimes ranging from 0.51 ms in AcN to 1.12 ms in DCM. These observations suggest that multiple solvent-dependent factors—such as vibrational quenching and stabilization of the excited state—may act simultaneously, with different contributions depending on the lanthanide ion. For Eu, electronic stabilization by the more polar AcN may outweigh vibrational deactivation, whereas for Tb, the reduced nonradiative decay in DCM appears to play a dominant role. In the solid state, the lifetime of 0.60 ms is in line with reported values for Tb^3+^ complexes bearing phenanthroline-type ligands [22,23].

The Sm-complex exhibits significantly shorter lifetimes (0.08–0.11 ms) across all media, which is typical for Sm^3+^ complexes due to the partially allowed nature of f–f transitions and lower intrinsic radiative rates. The minimal solvent dependence suggests dominant non-radiative decay pathways and weak solvent effects. Nonetheless, the detection of measurable Sm^3+^ emission in all media confirms successful energy transfer from the ligand, yet with limited efficiency.

The Dy complex exhibits the shortest luminescence lifetimes among the studied complexes, ranging from 0.0087 ms in DCM to 0.0114 ms in the solid state. These values reflect the inherently low radiative rates of dysprosium(III) and the dominance of multiphonon relaxation, especially given the small energy gap between the ^4^F_9⁄2_ emitting level and the vibrationally active ^6^H_j_ ground-state multiplets. The minimal variation in lifetime across media suggests limited influence of solvent polarity, which aligns with prior observations on phenanthroline-type Dy^3+^ complexes showing similarly weak emission profiles and solvent-insensitive behavior [17]. The detection of the characteristic ^4^F_9⁄2_ → ^6^H_15⁄2_ and ^6^H_13⁄2_ transitions, along with a yellow-to-blue intensity ratio (Y/B) exceeding 4, indicates a markedly asymmetric coordination environment, likely imposed by the steric and electronic effects of the para-methylphenoxyl substituent. Although the overall luminescence efficiency of Dy is modest (*Φ* ≈ 0.3%), the ligand effectively supports both ligand-to-metal energy transfer and coordination asymmetry. These combined features make Dy a viable candidate for magneto-optical applications that exploit both luminescence and magnetic behavior, as previously proposed for related systems [24].

#### 2.3.5. Fluorescence Titration and Binding Analysis

To complement the steady-state and time-resolved photophysical studies, fluorescence titrations were conducted to determine the thermodynamic parameters of complex formation between the complex and the trivalent lanthanide ions Eu^3+^, Tb^3+^, Sm^3+^, and Dy^3+^ in acetonitrile.

**Figure 12:**
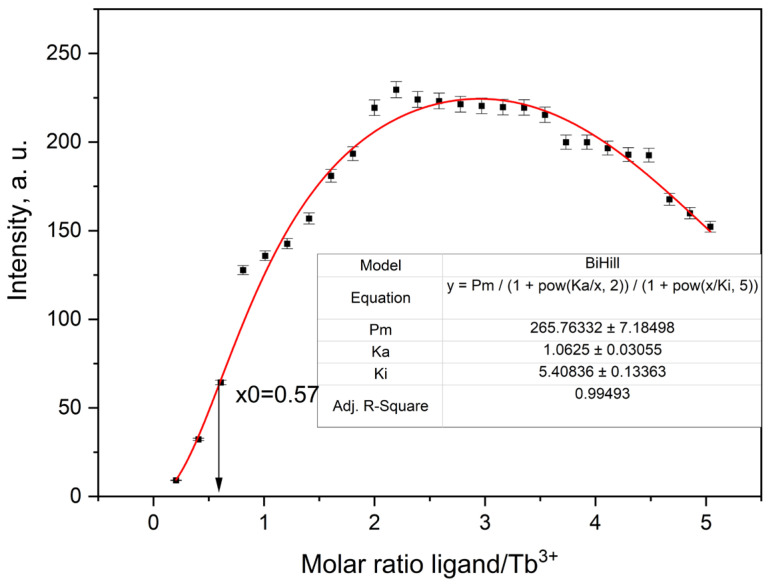
Fluorescence titration curve of Tb-complex in AcN.

The titration curves exhibited characteristic changes in emission intensity upon addition of the ligand, consistent with complex formation. Figure 12 shows the fluorescence titration curve for the Tb^3+^–ligand system, in which the emission intensity increases sharply with ligand addition, reaching a maximum near a 2:1 ligand-to-metal molar ratio. Data fitting using a fixed 2:1 ligand-to-metal stoichiometry yielded satisfactory correlation for all metal ions. The observed binding affinities followed the trend: Tb^3+^ > Eu^3+^ ≈ Dy^3+^ > Sm^3+^. This trend is broadly consistent with lanthanide contraction and the corresponding variations in ionic radius and ligand affinity [25]. The weaker binding observed for Sm^3+^ is consistent with its larger ionic radius and lower effective charge density, which reduce coordination strength. The stronger binding observed for Tb^3+^ may reflect more optimal size complementarity and coordination geometry within the ligand framework.

Overall, the titration results support the formation of well-defined 2:1 complexes in solution and highlight the ability of the ligand to discriminate subtly between different lanthanide ions. In all cases, the fluorescence intensity increased progressively with ligand addition, reaching a plateau near a 2:1 ligand-to-metal molar ratio, followed by moderate quenching. This behavior is characteristic of the formation of bis-ligand complexes of the type [Ln(ligand)_2_]^3+^. The titration data were analyzed using the BiHill model with fixed stoichiometry (*n* = 2), allowing determination of association constants (K_a_) and inhibition constants (K_i_), which reflect ligand affinity and possible competitive or saturation effects (Table 3). In this model, the inflection points of the titration curve—corresponding to the maximum rate of emission increase—can appear before the theoretical 2:1 ligand-to-metal ratio. This shift is characteristic of cooperative binding behavior, where the binding of the first ligand facilitates faster association of the second. Thus, the inflection observed at approximately 0.57 equivalents of ligand per metal ion supports the idea of stepwise complex formation, where binding of the first ligand enhances the likelihood of the second. This behavior is consistent with positive cooperativity in the formation of [Ln(ligand)_2_]^3+^ species. Overall, the titration results support the formation of well-defined 1:2 complexes in solution and highlight the ability of the ligand to discriminate subtly between different lanthanide ions.

**Figure 13:**
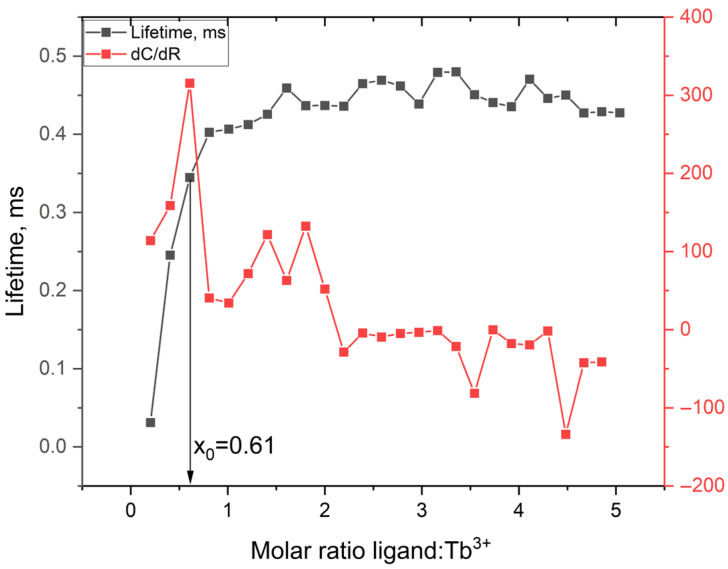
Fluorescence lifetime and the first derivative of emission intensity with respect to the ligand:Tb^3+^ molar ratio.

To experimentally verify this transition, the first derivative of the fluorescence intensity with respect to the ligand-to-metal ratio was calculated. The resulting curve (Figure 13) exhibited a distinct maximum at ≈0.61, which is in good agreement with the model-derived inflection point. The small deviation (about 7%) is well within the range of experimental errors and supports the reliability of the fitted model.

Time-resolved fluorescence measurements complemented the steady-state data, showing a clear increase in lifetime with increasing ligand concentration. The lifetime rose steeply between 0.2 and 1.0 equivalents and reached a plateau near 1.2 equivalents, reflecting stabilization of the Tb^3+^ excited state as ligand coordination progresses. Although full 2:1 complexation is expected based on stoichiometry, the earlier saturation in lifetime is attributed to rapid and cooperative formation of [Tb(ligand)_2_]^3+^ species. In this range, most of the emissive population appears to be formed prior to the theoretical endpoint.

The term “most emissive complexes” refers to the dominant, solution-stable [Tb(ligand)_2_]^3+^ species that account for the observed luminescence plateau. The use of the specific formula [Tb(ligand)_2_]^3+^ reflects the best-supported species based on emission behavior, titration inflection, and stoichiometric trends. For broader generality, one may alternatively denote these species as [Tb(ligand)ₙ]^3+^, where n approaches 2 before full saturation.

The lifetime data were acquired independently of the BiHill model, and their overlap with the intensity inflection region suggests that the main complexation event occurs within this concentration range. Here, “key” refers to the principal transition point at which coordination becomes sufficient to produce the dominant luminescent species. The inflection point—defined as the concentration at which the rate of emission increase (dC/dR) is maximal—marks the zone of most rapid complex formation. This implies that less emissive or partially coordinated species may dominate prior to this point.

These results indicate that complex formation likely proceeds through stepwise ligand association with positive cooperativity. The convergence of steady-state titration, lifetime saturation, and model-derived parameters (e.g., elemental analysis, R_0_, χ^2^ values) supports the efficient formation of [Tb(ligand)_2_]^3+^ species.

One may further speculate that the local maxima observed in the 1.5–2.0 R region of the dC/dR curve (Figure 13) reflect intermediate binding states within a stepwise mechanism. These secondary features may correspond to slower or partially reversible steps beyond the primary cooperative transition.

The observed lifetime increase could also be influenced by reduced vibrational quenching, improved ligand-to-metal energy transfer efficiency, or shielding of the metal ion from solvent quenchers. Elucidating these contributions would require additional structural insights (e.g., from NMR, EXAFS, or computational modeling) to better understand the coordination dynamics near the critical R values.

**Figure 14:**
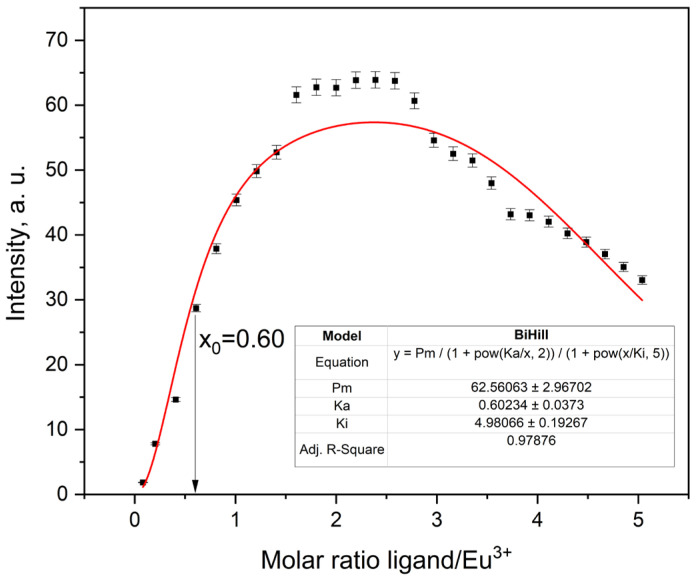
Fluorescence titration curve of Eu complex in AcN.

For Eu^3+^, the titration profile also shows a sharp increase in emission with ligand addition, reaching a maximum close to the expected 2:1 ratio (Figure 14). The fitted association constant was K_a_ = 0.60 ± 0.04. Slight fluctuations in the emission plateau led to a higher reduced χ^2^ (45.3), likely due to low signal variance, which accentuates small deviations from the model. Still, the model-derived inflection point (x_0_ = 0.60) matched the experimental first derivative maximum (x_0_ ≈ 0.61, Figure 12), providing strong support for the fitted cooperative 1:2 binding mechanism.

**Figure 15:**
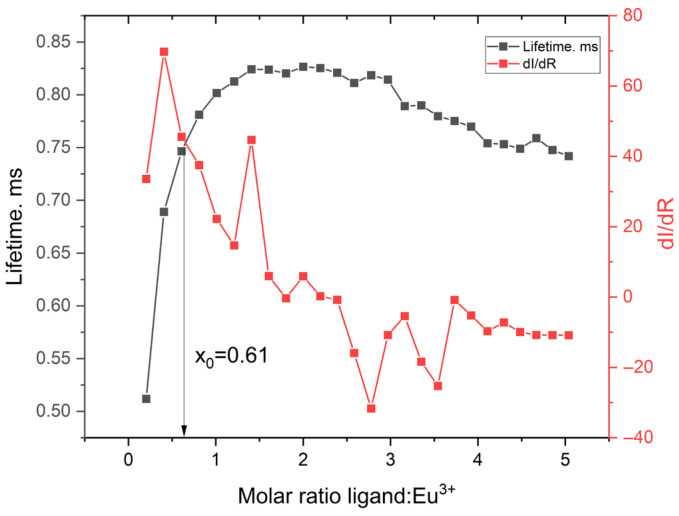
Fluorescence lifetime and the first derivative of emission intensity with respect to the ligand:Eu^3+^ molar ratio.

When directly compared, the lifetime and intensity derivative curves for both Tb^3+^ and Eu^3+^ complexes exhibit highly similar inflection points at x_0_ ≈ 0.61 (Figure 15). This consistency between two spectroscopically distinct lanthanides reinforces the robustness of the 1:2 binding model applied to the ligand. While the theoretical 2:1 stoichiometry corresponds to full ligand saturation, the inflection at ~0.6 equivalents reflect the point of maximal response, where the second ligand binds more readily following the first. This behavior is characteristic of positively cooperative, stepwise complex formation. Although the inflection point alone does not mathematically prove a 1:2 stoichiometry, its agreement with the fitted model and plateau near a 2:1 ratio supports the assignment. The comparison also highlights the value of using both steady-state and time-resolved data to resolve cooperative binding behavior.

**Figure 16:**
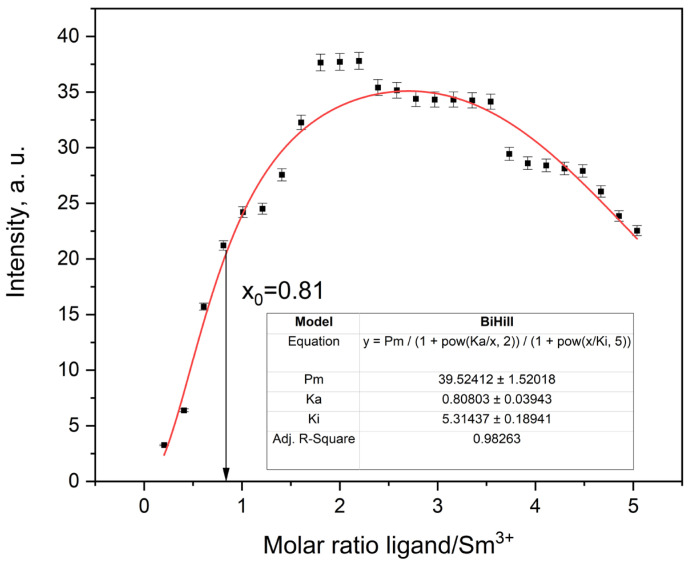
Fluorescence titration curve of Sm^3+^ in AcN.

The Sm^3+^ complex showed a more gradual increase in fluorescence intensity, yielding a smooth sigmoidal curve with a plateau near 2:1 ligand-to-metal ratio (Figure 16). The BiHill fixed model produced a satisfactory fit with an inflection at x_0_ = 0.81 and K_a_ = 0.81 ± 0.04. This delayed inflection, relative to Tb^3+^ and Eu^3+^, reflects the lower affinity of Sm^3+^, in line with its larger ionic radius and lower effective charge. At ligand-to-metal ratios above 2.0, a slight decrease in emission was observed (K_i_ = 5.31 ± 0.19). In this context, ligand over-saturation refers to excess ligand coordinating beyond the optimal 1:2 complex, potentially leading to weaker emissive species or competing equilibria involving higher-order adducts.

Notably, similar quenching behavior at R > 2 is also seen in the Tb^3+^ and Eu^3+^ systems, though it is less pronounced. This suggests a general trend across the series, where secondary ligand interactions or steric congestion may reduce overall emissive efficiency beyond the 2:1 point. While time-resolved data for Sm^3+^ were not available, the steady-state titration profile remains consistent with stepwise formation of [Sm(ligand)_2_]^3+^ under moderately cooperative conditions.

**Figure 17:**
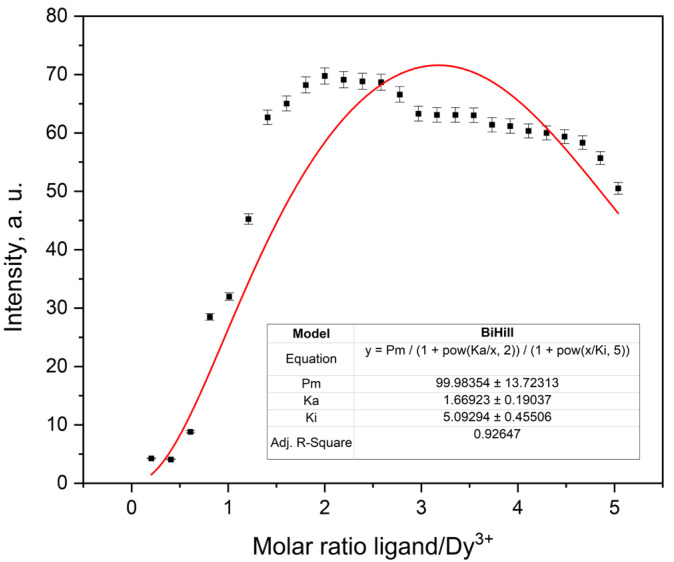
Fluorescence titration curve of Dy^3+^ in AcN.

Dy^3+^ followed a similar titration trend (Figure 17), with sigmoidal intensity growth and a plateau near the expected 2:1 ligand-to-metal ratio. However, fluctuations in the plateau region were more pronounced than in the other complexes, and deviations from the fitted model appear to begin already around R ≈ 1. Even so, the BiHill fixed model provided a reasonable fit (R^2^ = 0.93), and the highest K_a_ value in the series was obtained (1.67 ± 0.19). The high reduced χ^2^ (137) likely reflects minor structural heterogeneity or the onset of secondary equilibria at higher ligand concentrations.

Although lifetime data for Dy^3+^ are not available, the steep sigmoidal rise and early saturation support a 1:2 complex formation pathway. While the available data suggests cooperative binding, the extent of cooperativity is harder to quantify compared to the other ions due to the broader transition region and plateau irregularities. Thus, the behavior may best be described as consistent with stepwise complex formation involving some degree of positive cooperativity.

The results confirm the formation of 2:1 ligand-to-metal complexes for all four lanthanide ions. The association constants follow the trend Dy^3+^ > Tb^3+^ > Sm^3+^ > Eu^3+^, which aligns with known variations in ionic radii and Lewis acidity. In contrast, the inhibition constants (K_i_ between 4.98 and 5.41) show limited variation, indicating comparable tendencies toward ligand-induced quenching.

The obtained K_a_ values (0.6–1.7) are consistent with those typically reported for lanthanide complexes with bidentate or macrocyclic ligands in aprotic solvents such as AcN. For example, Eu^3+^ and Tb^3+^ complexes with bipyridine- and DOTA-derived ligands exhibit similar values under analogous conditions [17,26]. The relatively lower K_a_ values observed for the nitrate complexes can be attributed to the competitive inner-sphere coordination of the nitrate anions, which occupy coordination sites on the lanthanide center and weaken the metal–ligand interactions [27]. In contrast, significantly higher K_a_ values (10^5^–10^10^) are typically reported for complexes with non-coordinating counterions such as ClO_4_^−^ or CF_3_SO_3_^−^ [28]. This competitive binding, along with the tendency of nitrate complexes to form neutral species in solution, likely accounts for the systematically lower K_a_ values measured in our system. Likewise, the K_i_ values correspond well with previously observed behavior for secondary ligand effects and quenching [26,29].

Overall, the consistency of the data and model fits supports the robustness of the experimental approach and confirms that the 2-(4-methylphenoxy)-1,10-phenanthroline ligand is a suitable ligand for forming stable, luminescent lanthanide complexes. All parameters were extracted from fluorescence titration curves fitted with the BiHill fixed model (*n* = 2) and are summarized in Table 3.

The luminescence lifetimes of the Eu complex were measured in acetonitrile (AcN), dichloromethane (DCM), and in the solid state at 298 K. The recorded values were 2.116 ms (±0.0815 ms) in AcN, 1.799 ms (±0.0024 ms) in DCM, and 1.314 ms (±0.0120 ms) in the solid state. The longest lifetime observed in AcN suggests efficient suppression of non-radiative decay processes, likely due to the solvent being polar yet only weakly coordinating. This helps preserve the inner coordination sphere of Eu^3+^, minimizing interactions with high-energy oscillators such as O–H or N–H bonds, and favoring radiative deactivation via the characteristic ^5^D_0_ → ^7^F_j_ transitions.

In contrast, the shorter lifetime in the solid state is consistent with increased non-radiative decay due to lattice vibrations and intermolecular interactions. DCM, being non-coordinating and less polar than AcN, supports a moderately long lifetime but allows for slightly more vibrational quenching, reflected in its intermediate τ value. The absolute quantum yields were *Φ* = (42.05 ± 0.089)% in AcN and *Φ* = (37.15 ± 0.09)% in DCM. These correlate well with the observed lifetimes and confirm that AcN provides a favorable balance between coordination stability and vibrational damping.

The calculated radiative and non-radiative rate constants support this interpretation. In AcN, k_r_ ≈ 198.7 s^−1^ and k_nr_ ≈ 273.0 s^−1^ (k_r_/k_nr_ = 0.73), while in DCM, kr increases slightly to 206.5 s^−1^, but k_nr_ rises to 348.5 s^−1^ (k_r_/k_nr_ = 0.59), indicating greater non-radiative contribution in the latter. The ligand satisfies Latva’s criterion [3] for Eu^3+^ sensitization, with experimentally and computationally determined T_1_–^5^D_0_ energy gaps of 7257 and 6355 cm^−1^, respectively. Emission spectral analysis further supports this picture. The ^5^D_0_ → ^7^F_J_ transitions appear at 593 nm (J = 1), 615 nm (J = 2), 648 nm (J = 3), and 680 nm (J = 4). The calculated intensity ratios R2 = 6.25 and R = 7.31 confirm a highly asymmetric coordination environment, with strong electric-dipole transitions dominating. The methylphenoxy substituent on the ligand likely disrupts centrosymmetry and enhances ligand field perturbation, consistent with literature observations for substituted phenanthrolines.

The Tb complex shows different behavior. Luminescence lifetimes were measured as 0.512 ms (±0.00045 ms) in AcN, 1.118 ms (±0.0124 ms) in DCM, and 0.598 ms (±0.00034 ms) in the solid state. The highest τ in DCM reflects minimized non-radiative decay due to weak solvent interactions with Tb^3+^ and preserved coordination geometry. In contrast, the reduced lifetime in AcN points to enhanced quenching, likely facilitated by the solvent’s polarity and possible differential stabilization of ligand-based excited states. These results illustrate that the photophysical behavior of lanthanide complexes is not universally transferable between ions, even when the same ligand is used. While such differences are known in lanthanide chemistry, the distinct lifetime patterns observed here reinforce that sensitization and non-radiative deactivation pathways are strongly ion-specific. The solid-state lifetime is again the shortest, consistent with enhanced vibrational relaxation through lattice modes.

Quantum yields followed the same trend: *Φ* = 68.15 ±0.30% in DCM and *Φ* = 45.24 ± 0.09% in AcN. Radiative and non-radiative rates were calculated using k_r_ = Φ/τ and k_nr_ = (1 − *Φ*)/τ. In DCM, k_r_ ≈ 610 s^−1^ and k_nr_ ≈ 317 s^−1^ (k_r_/k_nr_ ≈ 1.92); in AcN, k_r_ ≈ 883 s^−1^ and k_nr_ ≈ 1067 s^−1^ (k_r_/k_nr_ ≈ 0.83). These results confirm that radiative processes dominate in DCM, while non-radiative decay is more pronounced in AcN. The T_1_—^5^D_4_ gap for Tb^3+^ is ≈2241 cm^−1^, which is slightly lower than the ideal range for efficient energy transfer. The observed photophysical behavior of Tb complex supports the conclusion that the ligand is a competent sensitizer. Energy transfer likely follows a Dexter-type mechanism [30], enabled by the bidentate coordination through the phenanthroline nitrogen atoms.

In the emission spectra, the ^5^D_4_ → ^7^F_5_ transition dominates at ≈545 nm, with clear splitting into Stark components indicating moderate ligand field asymmetry. The intensity ratio I(^5^D_4_ → ^7^F_5_)/I(^5^D_4_ → ^7^F_6_) serves as a qualitative symmetry probe, confirming distortion consistent with the structural features of ligand.

The Sm and Dy complexes showed moderate photophysical performance. The Sm complex achieved *Φ* = 2.84% (DCM) and 2.41% (AcN), with τ = 0.111 ms and 0.105 ms, respectively. These values surpass typical Sm^3+^ emitters in non-aqueous media, due to the rigid, conjugated ligand structure and electron-donating substituent. However, k_r_/k_nr_ ratios remain low (0.029 in DCM, 0.024 in AcN), confirming dominant non-radiative deactivation, a known challenge in Sm^3+^ photo physics.

For the Dy complex, steady-state emission spectra show the characteristic ^4^F_9⁄2_ → ^6^H_13⁄2_ (570 nm) and ^6^H_15⁄2_ (480 nm) transitions. The yellow-to-blue (Y/B) intensity ratio of 4.00 in DCM and 2.86 at 77 K indicates a strongly asymmetric coordination environment with enhanced electric-dipole character [31]. Absolute quantum yields were determined as 0.34 in DCM and 0.32 in AcN, consistent with literature values for phenanthroline-sensitized Dy^3+^ complexes [24]. Time-resolved measurements revealed very short excited-state lifetimes: 9.43 µs in DCM and 9.76 µs in AcN. These values reflect the intrinsic limitations of Dy^3+^ photophysics, where efficient non-radiative deactivation dominates due to small energy gaps and high vibrational coupling [32]. The calculated rate constants support this interpretation: in DCM, k_r_ ≈ 36.06 s^−1^ and k_nr_ ≈ 69.99 s^−1^ (k_r_/k_nr_ = 0.52); in AcN, k_r_ ≈ 32.79 s^−1^ and k_nr_ ≈ 69.67 s^−1^ (k_r_/k_nr_ = 0.47). Although radiative efficiency is modest, the emission is measurable and reproducible, enabled by the rigid and hydrophobic environment imposed by the ligand. Despite the limited emission efficiency of Dy^3+^, the data confirm that the ligand enables measurable ligand-to-metal energy transfer, even under unfavorable energetic conditions. The full set of photophysical parameters for all Ln complexes is shown in Table 4 for comparison.

To further validate the coordination environment and symmetry-related effects inferred from room-temperature data, low-temperature emission measurements were performed. Low-temperature (77 K) emission spectra across all Ln complexes reveal enhanced spectral resolution, including multiple Stark components, confirming low-symmetry ligand fields and reduced non-radiative decay. For the Eu complex, the ^5^D_0_ → ^7^F_2_ transition is split into five lines, and a weak ^5^D_0_ → ^7^F_0_ band appears around 580 nm, supporting the lower symmetry of the coordination polyhedral around the central metal ion. Similar Stark splitting patterns were observed for Tb, Sm, and Dy complex, confirming structural distortion across the series. These features reinforce the room-temperature findings and highlight the role of the para-substituted phenoxyphenanthroline scaffold in enforcing asymmetric coordination, minimizing vibrational quenching, and enabling efficient energy transfer.

## 3. Materials and Methods

All reagents were of analytical grade and used without further purification unless stated otherwise. Acetonitrile and dichloromethane (HPLC grade, ≥99.9%) were supplied by Fisher Chemicals, Shanghai, China. N-Methyl-2-pyrrolidone (NMP) was obtained from Merck (Darmstadt, Germany). 4-methylphenol and potassium carbonate were purchased from local distributors; the carbonate was dried gently in air and ground before use. 1,10-Phenanthroline monohydrate (99+%, Alfa Aesar, China Chemie GmbH, Beijing, China) was used as received. The synthesis of 2,4,6-trimethylphenol and 2-chloro-1,10-phenanthroline followed literature procedures [33,34]

Lanthanide nitrate hexahydrates (Ln = Tb, Eu, Sm, Dy) were prepared from the corresponding oxides (99.99%, Sigma–Aldrich, Milwaukee, WI, USA) by dissolution in dilute nitric acid (67%), followed by recrystallization of the resulting salts, as described in the literature [35].

Infrared spectra were collected on a Nicolet 6700 FT-IR spectrometer (Thermo Scientific, Dreieich, Germany) using KBr pellets. UV–Vis spectra were recorded in acetonitrile on a Thermo Scientific Evolution 300 spectrophotometer (Thermo Scientific, Dreieich, Germany).

Steady-state and time-resolved emission measurements were carried out in solution using a Horiba Jobin Yvon FluoroLog-3 fluorometer (Lyon, France). Absolute quantum yields were determined with a Quanta-ϕ F-3029 integrating sphere (150 mm diameter) using the formula:

*Φ* = (E_c_ − E_a_)/(L_a_ − L_c_)

where E_c_ and E_a_ are the integrated emission signals with and without the sample, and L_a_ and L_c_ refer to the excitation intensities.

Single-crystal X-ray diffraction was performed on Bruker D8 VENTURE (Bruker, Karlsruhe, Germany) or Rigaku XtaLAB Synergy-S diffractometers (Rigaku, Neu-Isenburg, Germany) with Mo Kα radiation (λ = 0.71073 Å). Structures were solved by direct methods using SHELXT [36] and refined with SHELXL2018 [37] within the ShelXle interface [38].

Elemental (CHN) analyses were performed on a Fisons Instruments EA 1108 elemental analyzer (Glasgow, Scotland, UK), according to the procedure recommended by the manufacturer.

Time-dependent density functional theory (TD-DFT) calculations were performed at the ωB97X-D/6-31G(d) level with the MWB52 effective core potential for Dy, as implemented in Gaussian 09W, Revision A.02 [39]

### 3.1. Synthesis of 2-(4-Methylphenoxy)-1,10-Phenanthroline

**Figure 18:**
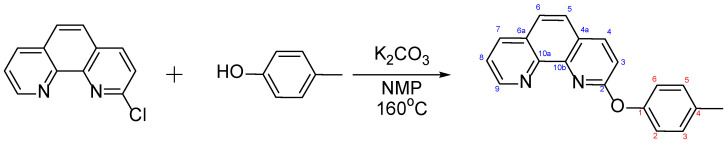
Chemical structure of 2-(4-methylphenoxy)-1,10-phenanthroline (C_19_H_14_N_2_O), showing carbon atom numbering for structural identification and NMR signal assignment.

In a flask, 2-chloro-1,10-phenanthroline (1.500 g, 7.00 mmol), 4-methylphenol (1.550 g, 14.33 mmol) and finely powdered anhydrous K_2_CO_3_ (2.897 g, 20.96 mmol) were suspended in N-methyl-2-pyrrolidone (10 mL, 103.9 mmol). The air in the flask was evacuated and the vessel was purged with argon. The resulting suspension was stirred at 160 °C for 24 h and after that the solvent was removed with water aspirator. The remaining crude product was dissolved in dichloromethane (DCM) (150 mL) and was washed with water (3 × 30 mL), aqueous NaOH (1 mol/L, 1 × 25 mL), and finally with water again (1 × 25 mL). The washed solution was dried over anhydrous Na_2_SO_4_, filtered, and the solvent was removed under reduced pressure. The brown solid residue was purified by flash chromatography on silica with hexanes-DCM (gradient elution). Yield-1.369 g (68%) pale-yellow solid. After recrystallization from ethyl acetate, the product was isolated in the form of white flakes. The chemical structure of the obtained ligand and the reaction equation are shown in Figure 18.

^1^H NMR δ 9.14 (dd, J = 4.3, 1.7 Hz, 1H, *H9-phenanthroline*), 8.22 (dd, J = 8.1, 1.7 Hz, 1H, *H7-phenanthroline*), 8.18 (d, J = 8.7 Hz, 1H, *H4-phenanthroline*), 7.75 (d, J = 8.7 Hz, 1H, *H5-phenanthroline*), 7.70 (d, J = 8.7 Hz, 1H, *H6-phenanthroline*), 7.59 (dd, J = 8.1, 4.3 Hz, 1H, *H8-phenanthroline*), 7.23 (d, J = 8.3 Hz, 2H, *H3,H5–4-methylphenoxy*), 7.18 (dt, J = 8.5, 2.1 Hz, 2H, *H2,H6–4-methylphenoxy*), 7.11 (d, J = 8.7 Hz, 1H, *H3-phenanthroline*), 2.39 (s, 3H, *CH_3_–4-methylphenoxy*). ^13^C NMR δ 162.85 (s, C2*-phenanthroline*), 152.41 (s, *^4^C10b-phenanthroline*), 150.21 (s, *C9-phenanthroline*), 145.43 (s, *^4^C1–4-methylphenoxy*), 145.19 (s, *^4^C10a-phenanthroline*), 139.77 (s, *C4-phenanthroline*), 135.82 (s, *C7-phenanthroline*), 134.29 (s, *^4^C4–4-methylphenoxy*), 130.44 (s, *C3,C5–4-methylphenoxy*), 129.09 (s, *^4^C6a-phenanthroline*), 125.86 (s, *C5-phenanthroline*), 125.35 (s, *^4^C4a-phenanthroline*), 124.67 (s, *C6-phenanthroline*), 122.90 (s, *C8-phenanthroline*), 120.67 (s, *C2,C6–4-methylphenoxy*), 112.29 (s, *C3-phenanthroline*), 20.87 (s, *CH_3_–4-methylphenoxy*). Rf TLC 0.72 (neutral Al_2_O_3_, DCM), 0.23 (silica, ethyl acetate). m.p. 189.4 °C. Elemental Analysis: Calculated: C, 79.70; H, 4.93; N, 9.78; O, 5.59. Found: 79.68; H, 4.95, N, 9.77; O, 5.60.

### 3.2. Synthesis of Lanthanide(III) Complexes

The synthesis procedure was adapted from a previously reported method for preparing 1,10-phenanthroline-based Eu(III) complexes [40]. Briefly, four lanthanide(III) complexes were synthesized by reacting the nitrate salts of the respective lanthanide ions (Ln(NO_3_)_3_·6H_2_O, where Ln = Tb, Eu, Sm, Dy) with the corresponding ligand in a 1:2 metal-to-ligand molar ratio in acetonitrile. The mixtures were heated at 80 °C for 7 h, followed by standing at room temperature for an additional 12 h. The resulting precipitates were collected by filtration, washed three times with acetonitrile, and dried in an oven at 60 °C. Yields for all four complexes were approximately 80%. Single crystals suitable for structural characterization were obtained by slow evaporation of saturated acetonitrile solutions at room temperature.

Elemental Analysis:

Calculated for [Dy(L24)_2_(NO_3_)_3_] (%): C, 49.55; H, 3.06; N, 10.64; O, 19.10;

Found for [Dy(L24)_2_(NO_3_)_3_] (%): C, 49.57; H, 3.08; N, 10.62; O, 19.07;

Calculated for [Eu(L24)_2_(NO_3_)_3_] (%): C, 50.12; H, 3.10; N, 10.77; O, 19.33;

Found for [Eu(L24)_2_(NO_3_)_3_] (%): C, 49.87; H, 3.13; N, 10.74; O, 19.31;

Calculated for [Sm(L24)_2_(NO_3_)_3_] (%): C, 50.21; H, 3.10; N, 10.79; O, 19.36;

Found for [Sm(L24)_2_(NO_3_)_3_] (%): C, 50.22; H, 3.13; N, 10.76; O, 19.33;

Calculated for [Tb(L24)_2_(NO_3_)_3_] (%): C, 49.74; H, 3.08; N, 10.69; O, 19.18;

Found for [Tb(L24)_2_(NO_3_)_3_] (%): C, 49.76; H, 3.12; N, 10.70; O, 19.21.

### 3.3. Photophysical Measurements

Photophysical studies were carried out to evaluate the ability of ligand and its lanthanide complexes to absorb and emit light across different environments. Key spectral features were examined in various solvents and in solid state, with particular focus on the excited-state behavior, fluorescence lifetimes, and the efficiency of ligand-to-metal energy transfer.

Special attention was given to the influence of solvent polarity and coordination geometry on emission characteristics and excited-state deactivation pathways. Low-temperature measurements were also performed to determine the ligand’s triplet-state energy and to assess its energetic alignment with the emissive levels of the respective lanthanide ions. The collected data provides insights into the sensitization mechanism and the role of structural factors in governing the luminescent properties of the complexes.

### 3.4. Fluorescence Titration Procedure

Fluorescence titrations were performed to study the complexation of the ligand with Eu^3+^, Tb^3+^, Sm^3+^, and Dy^3+^ in acetonitrile. Stock solutions of the ligand (1.0 mM) and lanthanide (III) nitrates (0.01 mM) were prepared in spectroscopic-grade acetonitrile. The titrations were carried out by incremental addition of the ligand solution to 2.45 mL of metal ion solution placed in a 1 cm path length quartz cuvette, under ambient conditions. After each addition, the solution was briefly mixed, and the emission spectrum was recorded using 295 nm excitation.

Emission maxima were monitored at 545 nm (Tb^3+^), 615 nm (Eu^3+^), 645 nm (Sm^3+^), and 570 nm (Dy^3+^). The total volume increase did not exceed 5%, and no dilution correction was applied. The titration data were fitted using a fixed 1:2 metal-to-ligand binding model. Curve fitting and numerical analysis, including first-derivative evaluation, were performed using OriginPro 2024b.

## 4. Conclusions

The para-substituted phenanthroline ligand L24 provides a rigid yet adaptable framework for forming stable lanthanide complexes. The 4-methylphenoxy group at the 2-position introduces slight asymmetry without affecting chelation, which promotes energy transfer and strengthens electric-dipole transitions, especially in Eu^3+^ and Tb^3+^ complexes. Sm^3+^ and Dy^3+^ complexes emit less efficiently but remain clearly luminescent in different solvents, showing that L24 supports ligand-to-metal sensitization even under less favorable energy conditions.

Fluorescence titrations confirmed positively cooperative 2:1 complex formation for all four lanthanides, with binding constants between log K = 0.60 and 1.67. The triplet-state energy of L24 (22,741 cm^−1^, 2.82 eV) matches the emissive levels of Eu^3+^ and Tb^3+^, enabling efficient energy transfer. The Tb^3+^ complex achieved a quantum yield of about 68% and an excited-state lifetime of 1.12 ms, while the Eu^3+^ complex reached 42% and 2.12 ms, respectively.

Modifying the phenanthroline core with a para-substituted aryl group effectively adjusts symmetry and controls sensitization pathways. L24 serves as a practical scaffold for creating luminescent lanthanide complexes suitable for diverse photonic applications.

## Figures and Tables

**Table 1:** Calculated adiabatic triplet-state energies (ΔET_1_) of the ligand and its lanthanide complexes.

Ion	ΔET_1_ (Ligand) [eV]	ΔET_1_ (Complex) [eV]	ΔET_1_ (Complex–Ligand) [eV]
Eu^3+^	2.92	2.82	−0.1
Tb^3+^	2.92	2.95	0.03
Sm^3+^	2.92	2.82	−0.1
Dy^3+^	2.92	3.16	0.24

**Table 2:** Fluorescence lifetimes (τ) of L24 complexes with Eu^3+^, Tb^3+^, and Sm^3+^ in different physical states and solvents, measured at room temperature.

Complex	State	τ (ms)
Eu	Dichloromethane	1.799 ± 0.0024
Eu	Acetonitrile	2.116 ± 0.0815
Eu	Solid	1.314 ± 0.012
Tb	Dichloromethane	1.118 ± 0.0124
Tb	Acetonitrile	0.512 ± 0.0004
Tb	Solid	0.598 ± 0.0003
Sm	Acetonitrile	0.105 ± 0.0004
Sm	Dichloromethane	0.111 ± 0.0001
Sm	Solid	0.080 ± 0.0003
Dy	Dichloromethane	0.009 ± 0.0003
Dy	Acetonitrile	0.010 ± 0.0009
Dy	Solid	0.011 ± 0.0001

**Table 3:** Association constants (K_a_), inhibition constants (K_i_), maximal emission intensities (P_m_), fit quality (R^2^), and reduced chi-square (χ^2^ red) values obtained from fluorescence titration of the ligand with Ln^3+^ ions in acetonitrile at 298 K (BiHill model, *n* = 2).

Metal Ion	P_m_ * (a.u.)	K_a_	K_i_	R^2^	χ^2^ Reduced
Tb^3+^	265.76332 ± 7.18498	1.0625 ± 0.03055	5.40836 ± 0.13363	0.99493	10.32388
Eu^3+^	62.56063 ± 2.96702	0.60234 ± 0.0373	4.98066 ± 0.19267	0.9804	45.25359
Sm^3+^	39.52412 ± 1.52018	0.80803 ± 0.03943	5.31437 ± 0.18941	0.98402	25.32979
Dy^3+^	99.98354 ± 13.72313	1.66923 ± 0.19037	5.09294 ± 0.45506	0.93235	136.9168

* P_m_ values are reported in relative units (a.u.) and primarily reflect the signal intensity under the experimental conditions. Due to differences in lanthanide emission efficiencies and spectral properties, these values are not strictly comparable across metal ions.

**Table 4:** Photophysical parameters of Ln complexes in different solvents and physical states.

Complex	*Φ* in Acetonitrile (%)	*Φ* in DCM (%)	τ in Acetonitrile (ms)	τ in DCM (ms)	τ in Solid (ms)
Eu	42.05 ± 0.09	37.15 ± 0.09	2.12 ± 0.08	1.80 ± 0.002	1.31 ± 0.012
Tb	45.24 ± 0.09	68.15 ± 0.30	0.51 ± 0.0004	1.12 ± 0.012	0.60 ± 0.0003
Sm	2.41 ± 0.03	2.84 ± 0.05	0.105 ± 0.0004	0.111 ± 0.0001	0.080 ± 0.0003
Dy	0.32 ± 0.02	0.34 ± 0.03	0.00976 ± 0.0013	0.00943 ± 0.0005	0.00997± 0.0001

## Data Availability

Data is contained within the article or Supplementary Materials. Further inquiries can be directed at the corresponding authors.

## Supplementary Materials

The following supporting information can be downloaded at: https://www.mdpi.com/article/10.3390/molecules30173548/s1, Figure S1: Powder XRD patterns of (a) the complexes and (b) the ligands compared with the simulated data from the single crystal diffraction measurements; Figure S2: H-bonds between adjacent ligand molecules; Figure S3: Emission spectra of (a) Eu, (c) Sm, (e) Tb and (g) Dy at 77K and (b) Eu, (d) Sm, (f) Tb and (h) Dy in solid state; Table S1: Crystal structure and refinement data of the obtained ligand and it’s Dy-complex [41]; Table S2: Continuous shape measurements of coordination polyhedral; Table S3: Summarized colorimetric data of the complexes.
