# Supplementary material for: A Para-Substituted 2-Phenoxy-1,10-Phenanthroline Ligand for Lanthanide Sensitization: Asymmetric Coordination and Enhanced Emission from Eu3+, Tb3+, Sm3+ and Dy3+ Complexes
Source: Molecules. 2025 Aug 29;30(17):3548. doi: 10.3390/molecules30173548 (PMC12430686; doi:10.3390/molecules30173548)
Supplement: Supplementary file 1 [file molecules-30-03548-s001.zip › molecules-3722585-supplementary.pdf]

A para-substituted 1,10-phenanthroline ligand for lanthanide sensitization: asymmetric coordination and enhanced emission from Eu^{3+}, Tb^{3+}, Sm^{3+} and Dy^{3+} complexes

Joana Zaharieva[1*], Vladimira Videva[2,3], Mihail Kolarski[2], Rumen Lyapchev[2], Bernd Morgenstern[4] and Martin Tsvetkov[1*]

[1]Laboratory of Chemistry of Rare-Earth Elements, Faculty of Chemistry and Pharmacy, Sofia University "St. Kliment Ohridski", 1164 Sofia, Bulgaria;

[2]Faculty of Chemistry and Pharmacy, Sofia University "St. Kliment Ohridski", 1164 Sofia, Bulgaria; ohtvv@chem.uni-sofia.bg (V.V), mkolarski@uni-sofia.bg (M.K.), ohrl@chem.uni-sofia.bg (R.L.)

[3]Institute of Optical Materials and Technologies, Bulgarian Academy of Sciences, 1113 Sofia, Bulgaria

[4]Inorganic Solid State Chemistry, Saarland University, Campus Geb. C4 1, 66123 Saarbrücken, Germany; bernd.morgenstern@uni-saarland.de (B.M)

**Correspondence: nhjz@chem.uni-sofia.bg (J.Z.); nhmt@chem.uni-sofia.bg (M.T.)*

Table of contents:

Figure S1. Powder XRD patterns of a) the complexes and b) the ligands compared with the simulated data from the single crystal diffraction measurements

Figure S2. Intermolecular H-bonds between two adjacent a) ligand and b) complex molecules

Figure S3. Emission spectra of a) Eu, c) Sm, e) Tb and g) Dy at 77K and b) Eu, d) Sm, f) Tb and h) Dy in solid state

Table S1. Crystal structure and refinement data of the obtained ligand and it's Dy-complex

Table S2. Continuous shape measurements of coordination polyhedral

Table S3. Summarized colorimetric data of the complexes

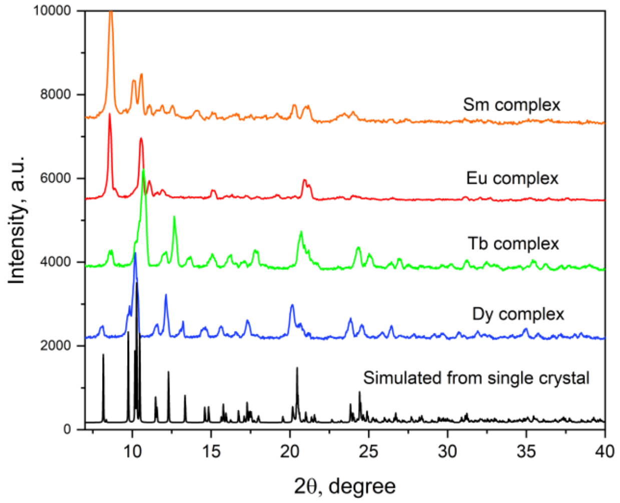

a

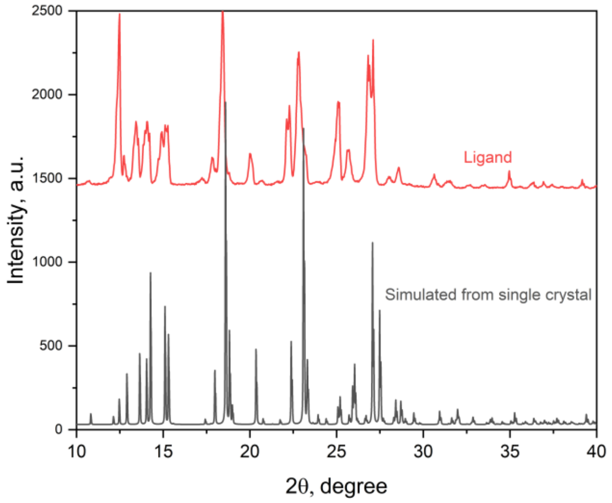

b

Figure S1. Powder XRD patterns of a) the complexes and b) the ligands compared with the simulated data from the single crystal diffraction measurements

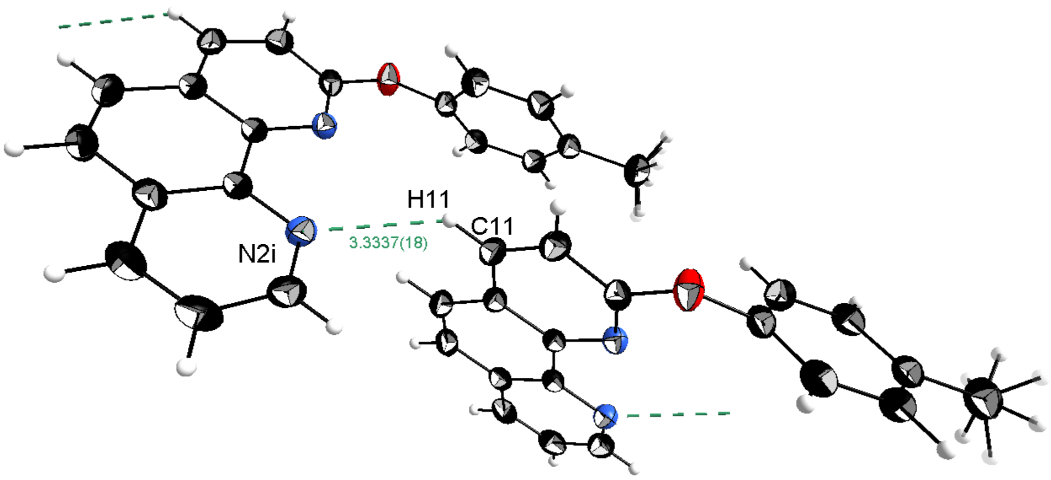

a

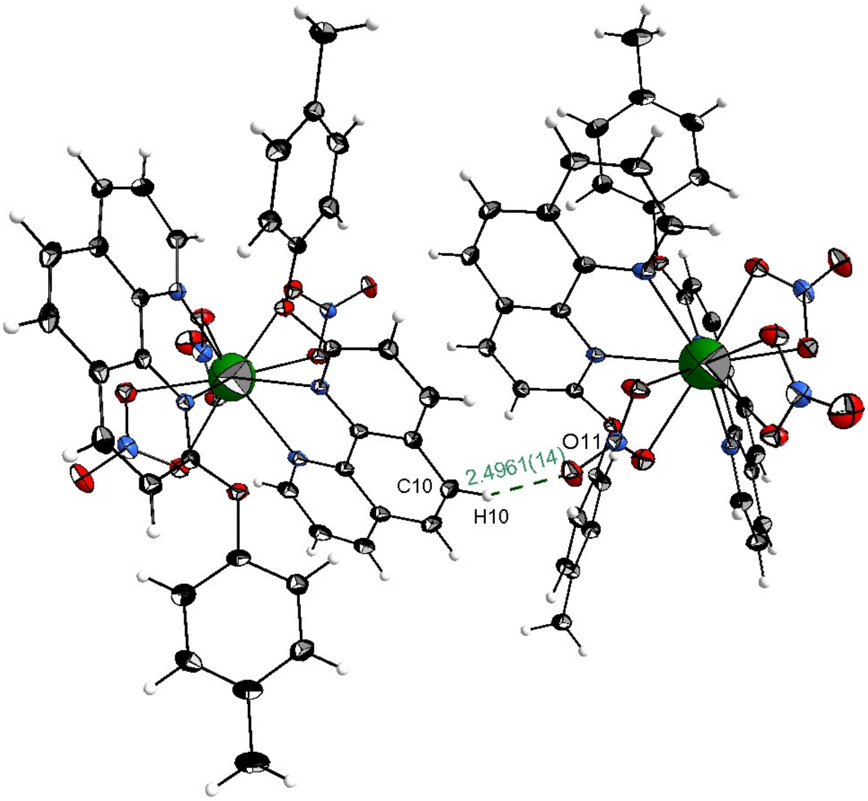

Figure S2. Intermolecular H-bonds between two adjacent a) ligand and b) complex molecules

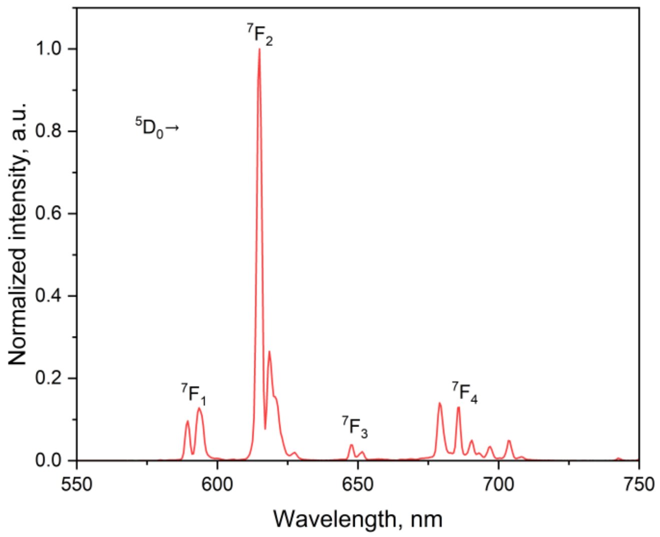

a

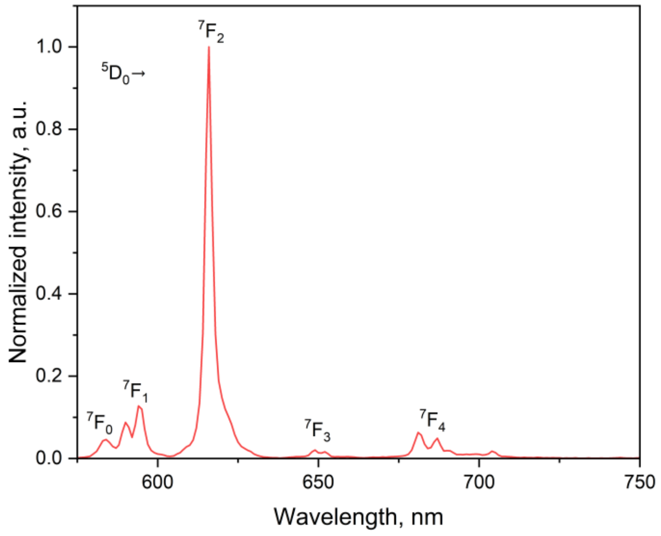

b

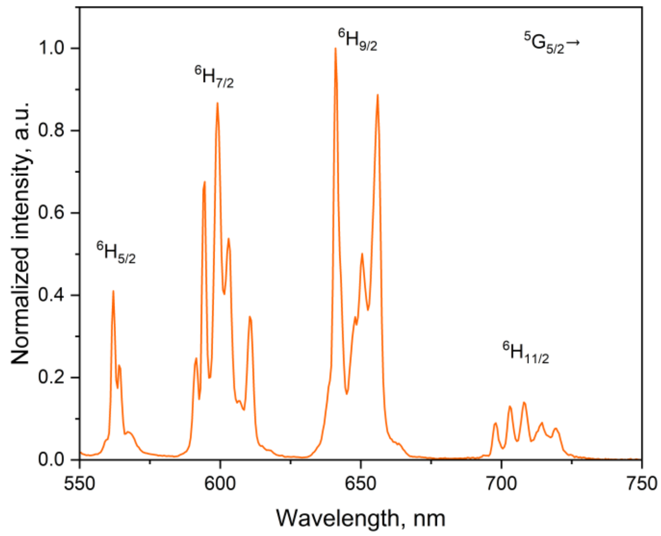

c

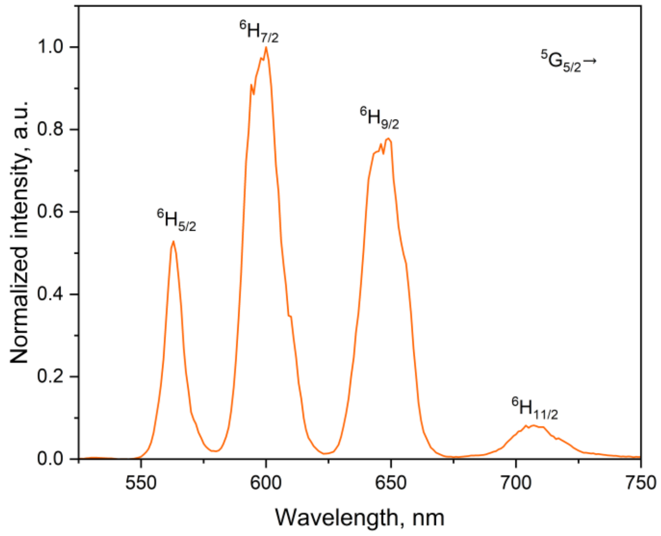

d

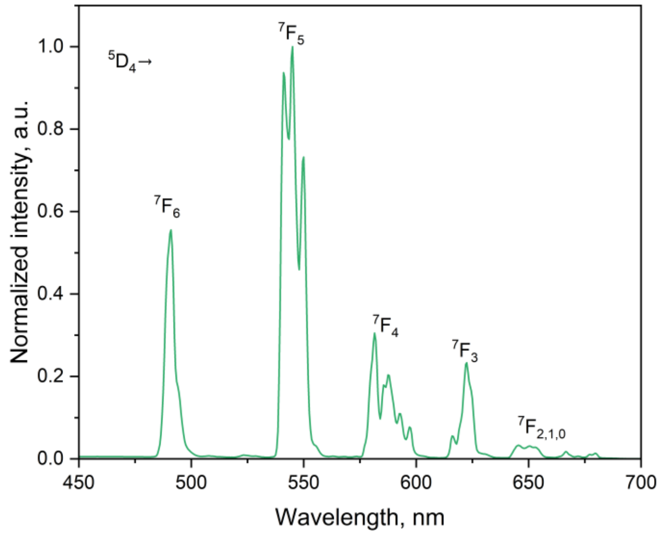

e

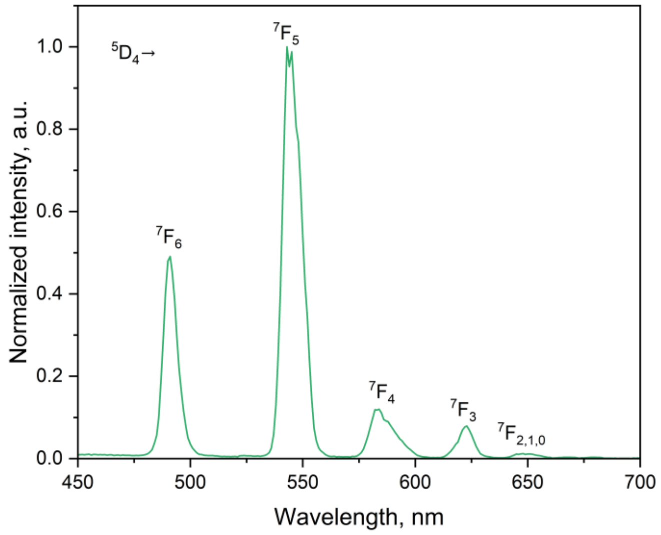

f

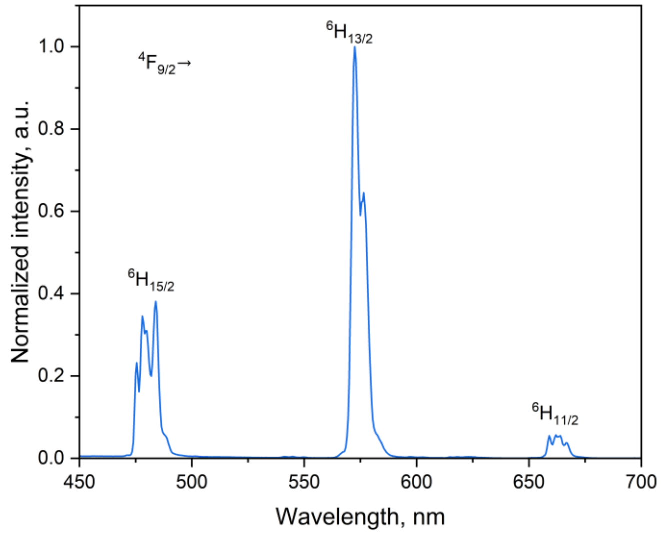

g

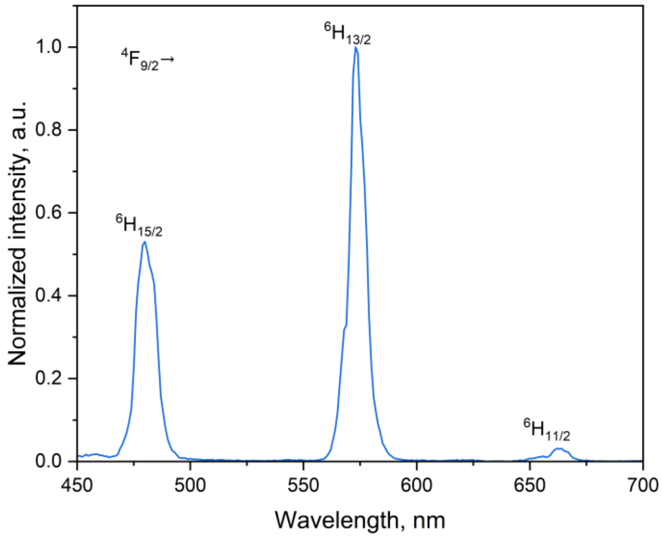

h

Figure S3. Emission spectra of a) Eu, c) Sm, e) Tb and g) Dy at 77K and b) Eu, d) Sm, f) Tb and h) Dy in solid state

Table S1. Crystal structure and refinement data of the obtained ligand and it's Dy-complex

	**Ligand**	**Dy-complex**
**CCDC numbers**	2457307	2457311
**Crystal data**		
**Empirical formula**	$C_{19}H_{14}N_2O$	$C_{38}H_{28}DyN_7O_{11}$
**Molecular weight, (g/mol)**	286.32	921.17
**Crystal system, space group**	Orthorhombic, *Pbca*	Triclinic, $P\bar{1}$
**Temperature (K)**	130	143
***a, b, c* (Å)**	14.1944(7), 13.7103(6), 14.5824(6)	11.1154(3), 11.3728(3), 14.3935(4)
**α, β, γ (°)**	90.0, 90.0, 90.0	90.241(1), 90.992(1) 107.689(1)
**V, (Å^3)**	2837.9(2)	1733.16(8)
**Z**	8	2
**Radiation type**	Mo Kα	Mo Kα
**μ, (mm^{-1})**	0.084	2.233
**Crystal size (mm)**	0.08 x 0.18 x 0.38	0.02 x 0.18 x 0.20
**Data collection**		
**Diffractometer**	Rigaku XtaLAB Synergy-S	Bruker D8 Venture
**Absorption correction**	multi-scan, spherical harmonics	Multi-scan, SADABS[1]
**T_{min}, T_{max}**	0.2892, 1.0000	0.6773, 0.7456
**No. of measured, independent and observed [I > 2σ(I)] reflections**	27947, 3134, 2481	68069, 8284, 7806
**R_{int}**	0.075	0.032
**$(\sin \theta/\lambda)_{max}$, (Å^{-1})**	0.6414	0.6586
**Refinement**		
**R[$F^2 > 2\sigma(F^2)$], wR(F^2), S**	0.0410, 0.1208, 1.08	0.0176, 0.0398, 1.09

**No. of reflections**	3134	8284
**No. of restrains**	0	0
**No. of parameters**	201	516
**H-atom treatment**	H-atom parameters constrained	H-atom parameters constrained
**$\Delta\rho_{max}$, $\Delta\rho_{min}$ (e Å^{-3})**	0.20, -0.25	0.39, -0.49

Table S2. Continuous shape measurements of coordination polyhedral

DP-10	EPY-10	OBPY-10	PPR-10	PAPR-10	JBCCU-10	JBCSAPR-10	JMBIC-10	JATDI-10	JSPC-10	SDD-10	TD-10	HD-10
26.998	22.056	15.087	12.955	11.143	14.315	16.217	10.337	18.903	13.837	11.131	11.937	11.835

IUPAC abbreviations for ten coordinated ions: DP-10= Decagon (D_{10d}), EPY-10=Enneagonal pyramide (C_{9v}), OBPY-10= Octagonal bipyramid (D_{8h}), PPR-10= Pentagonal prism (D_{5h}), PAPR-10=Pentagonal antiprism (D_{5h}), JBCCU-10= Bicapped cube J15 (D_{4h}), JBCSAPR-10= Bicapped square antiprism J17 (D_{4h}), JMBIC-10=Metabidiminished icosahedron J62 (C_{2v}), JATDI-10=Augmented tridiminished icosahedron J64 (C_{3v}), JSPC-10= Sphenocorona J87 (C_{2v}), SDD-10= Staggered Dodecahedron (2:6:2) (D_2), TD-10= Tetradecahedron (2:6:2) (C_{2v}), HD-10=Hexadecahedron (2:6:2) or (1:4:4:1) (D_{4h})

Table S3. Summarized colorimetric data of the complexes

Metal Ion	x-, y- coordinates	CCT, K	λ_{dom}, nm	Color Purity, %
Sm	0.58427, 0.39705	1712.1	594.2	94.6
Eu	0.6279, 0.36128	2088.9	602.4	96.9
Tb	0.29421, 0.59835	6173.3	546.6	69.5
Dy	0.38676, 0.43107	4157.1	570.9	45.5

References:

1. Krause, L.; Herbst-Irmer, R.; Sheldrick, G.M.; Stalke, D. Comparison of Silver and Molybdenum Microfocus X-Ray Sources for Single-Crystal Structure Determination. *J Appl Crystallogr* 2015, *48*, 3–10, doi:10.1107/S1600576714022985.
